# A life span developmental investigation of marriage and problem-drinking reduction

**DOI:** 10.1017/S0954579422000827

**Published:** 2022-10-26

**Authors:** Matthew R. Lee, Ellen W. Yeung, Andrew K. Littlefield, Audrey Stephenson, Annabel Kady, Thomas Kwan, Laurie Chassin, Kenneth J. Sher

**Affiliations:** 1 Department of Applied Psychology, Center of Alcohol Studies, Rutgers University, Piscataway, NJ, USA; 2 Department of Psychological and Brain Sciences, The George Washington University, Washington, DC, USA; 3 Department of Psychological Sciences, Texas Tech University, Lubbock, TX, USA; 4 Department of Psychology, Arizona State University, Tempe, AZ, USA; 5 Department of Psychological Sciences, University of Missouri, Columbia, MO, USA

**Keywords:** alcohol use disorder, desistance, lifespan development, marriage, maturing out, natural recovery, problem drinking

## Abstract

While prior literature has largely focused on marriage effects during young adulthood, it is less clear whether these effects are as strong in middle adulthood. Thus, we investigated age differences in marriage effects on problem-drinking reduction. We employed parallel analyses with two independent samples (analytic-sample *N*s of 577 and 441, respectively). Both are high-risk samples by design, with about 50% of participants having a parent with lifetime alcohol use disorder. Both samples have been assessed longitudinally from early young adulthood to the mid-to-late 30s. Separate parallel analyses with these two samples allowed evaluation of the reproducibility of results. Growth models of problem drinking tested marriage as a time-varying predictor and thereby assessed age differences in marriage effects. For both samples, results consistently showed marriage effects to be strongest in early young adulthood and to decrease somewhat monotonically thereafter with age, reaching very small (and nonsignificant) magnitudes by the 30s. Results may reflect that role transitions like marriage have more impact on problem drinking in earlier versus later adulthood, thereby highlighting the importance of life span developmental research for understanding problem-drinking desistance. Our findings can inform intervention strategies aimed at reducing problem drinking by jumpstarting or amplifying natural processes of adult role adaptation.

## Introduction

Problem drinking has myriad personal, public health, and economic costs (NIAAA, 2020; Sher & Vergés, 2016). Normative declines in problem drinking are observed to occur beginning in early young adulthood and continuing throughout the life span (Lee & Sher, 2018; Schulenberg et al., 2019). Despite this age-related trend toward problem-drinking reduction, some individuals exhibit chronic problem drinking well into adulthood. Given this developmental heterogeneity, research should aim to illuminate factors that differentiate developmentally limited versus chronic adulthood problem drinking. Findings can guide intervention strategies from a life span developmental perspective (NIAAA, 2017).

### Life span development and marriage effects

Robust evidence suggests that family role transitions like marriage and parenthood are important influences on young–adult drinking reductions, including desistance from alcohol use disorder (AUD) (as reviewed by Lee & Sher, 2018). The “maturing out” literature (O’Malley, 2004) has often interpreted such family role effects through role incompatibility theory (Yamaguchi & Kandel, 1985). For instance, demands of a new marital role may conflict with prior established drinking behaviors, prompting drinking-related reductions to resolve the conflict. In role incompatibility theory, this adaptation to reduce role conflict is termed “role socialization.”

As marriage effects are primarily associated with young–adult “maturing out,” questions remain regarding the extent these effects also occur at later ages. There is reason to suspect that marriage may exert particularly strong influences in young adulthood. Established developmental theory (e.g., Erikson, 1968) views entry into adult roles like marriage to be a key young–adult developmental task. Further, more general arguments have been made that environmental effects on behavior may decrease throughout adulthood as age brings a greater tendency for adults to actively construct their environments to align with personal characteristics (as opposed to more passively receiving and then reacting/adapting to their environments; Kendler et al., 2007; Scarr & McCartney, 1983). Thus, after young adulthood, there may be a decreased tendency for role socialization whereby individuals enter marriage and then adapt to reduce role conflict. In contrast, there may be an increased tendency for “role selection,” for example, with individuals actively avoiding or pursuing marriage depending on the extent that their personal characteristics (e.g., drinking behaviors) can be expected to cause role conflict. The above arguments for ways that different role incompatibility-related processes may shift across the adult life span provide a conceptual basis for the current study’s prediction: effects of marriage on problem-drinking reductions will be strongest in young adulthood and decrease thereafter with age. Such questions align with a broader empirical objective of understanding differences across the adult life span in factors that influence problem-drinking reduction.

Some past studies of marriage effects on drinking-related reductions have shown weaker, nonsignificant, or even opposite (i.e., risk-conferring) effects at older ages (Bennett et al., 1999; Prescott & Kendler, 2001); others have shown either little evidence of age differences or yielded inconsistent findings (Neve et al., 2000; Temple et al., 1991). Limitations of past studies have included contrasts of broad age groups (e.g., 18–39 vs. 40+), failure to test age moderation, analysis of cross-sectional associations that obscure directionality, and prediction of low severity drinking outcomes with limited clinical relevance.

To address these limitations, the current study conducted a focused investigation of age differences in marriage effects with nuanced age contrasts, formal tests of age moderation, longitudinal analyses of prospective marriage effects, and prediction of an outcome designed to capture both subclinical and clinical variation in problem drinking. Further, we tested this question in two independent samples to internally assess replicability of findings.

## Method

### Participants and procedures

This study involved separate analyses of data from two independent samples from the Adult and Family Development project (AFDP; Chassin et al., 1992) and the Alcohol, Health, and Behavior project (AHB; Sher et al., 1991), respectively. Both are high-risk samples over-representing familial AUD (~50% of participants had a parent with lifetime AUD [see below]). Both samples have been assessed longitudinally from early young adulthood to the mid-to-late 30s (see Table 1 and Supplemental Appendix S1). Analyses excluded those who were alcohol abstainers across all waves used in the current study (AFDP *n* = 72; AHB *n* = 7). Analyses also excluded those who were already married at the first analytic wave (AFDP *n* = 195; AHB *n* = 26), given our interest in longitudinal transitions from being unmarried to married. These exclusion criteria resulted in analytic samples of *n* = 577 for AFDP and *n* = 441 for AHB.

### AFDP participants

Initially recruited as adolescents in the mid-1980s as part of a multigenerational study of familial risk (*N* = 454; *M*_age_= 12.7; *SD*_age_= 1.45; 53% male; 72% non-Hispanic Caucasian; Chassin et al., 1992, 1991), 54% of AFDP participants had a parent with lifetime AUD. Potential high-risk families were identified using court records for intoxicated driving, health maintenance questionnaires, and community telephone screenings. Telephone screening also identified potential low-risk families matched to high-risk families on ethnicity, family structure, adolescent’s age, and socioeconomic status. Computerized structured interviews assessed DSM-III parental lifetime alcohol abuse or dependence (American Psychiatric Association, 1980; DIS-III; Robins et al., 1981, 1982). Retained high-risk families had at least one biological, custodial parent who met lifetime criteria for alcohol abuse or dependence. Retained low-risk families had no parents who met lifetime criteria for alcohol abuse or dependence. For more recruitment details, see Chassin et al. (1992).

After initial recruitment, three adolescent waves of data were collected annually (Waves 1–3; mean ages of 13.22 [SD = 1.47], 14.17 [SD = 1.46], and 15.16 [SD = 1.47]). Five years later, at Wave 4, full-biological siblings were recruited into the sample if they were within the same age range as the original participants (*n* = 390; resulting in *N* = 844 with 192 sets of two siblings, 63 sets of 3 siblings, and 22 sets of 3 or more siblings). Three adulthood waves were then collected at 5-year intervals (Waves 4–6; mean ages of 21.1 [SD = 2.3], 27.1 [SD = 3.4], 33.4 [SD = 3.6]). The current study used only the adulthood AFDP data from Waves 4–6. Longitudinal retention consistently exceeded 90% at Waves 4–6 for both original participants and siblings.

### AHB participants

Initially recruited as college freshmen at a large Midwestern University in 1987 (*N* = 489; *M*_age_= 18.6; *SD*_age_= 0.97; 53% male; 93% non-Hispanic Caucasian; Sher et al., 1991), 48% of AHB participants had a father with lifetime AUD. At the start of this study, 3156 potential participants were screened for parental problem drinking using an adapted version of the Short Michigan Alcoholism Screening Test (Crews & Sher, 1992; Selzer et al., 1975). Of these respondents, 808 were tentatively classified as either high or low risk and then interviewed using sections of the Family History-Research Diagnostic Criteria (FH-RDC; Endicott et al., 1978). Respondents were retained in the final high-risk group if the FH-RDC confirmed paternal AUD (regardless of maternal AUD). Respondents were retained in the final low-risk group if the FH-RDC detected no first-or-second-degree relatives with AUD or drug use disorder and detected no first-degree relatives with antisocial personality disorder. For recruitment details, see Sher et al. (1991).

After initial recruitment, four waves of data were collected annually during college (Waves 1–4; ages 18, 19, 20, and 21), and then three post-college waves were collected over a subsequent span of 14 years (Waves 5–7; ages 25, 29, and 35). The current study used only the AHB data from Waves 4–7, as transitions to marriage were rare in the earlier waves. Retention of original participants at Waves 4–7 was 96%, 93%, 83%, and 78%, respectively.

### Measures

#### Problem drinking

A count of past-year drinking consequences (e.g., physical fights, complaints from others) and alcohol-dependence symptoms (e.g., loss of control, tolerance, withdrawal; per Edwards & Gross’s [1976] dependence syndrome) was derived based in part on items from the Michigan Alcoholism Screening Test (Selzer, 1971). The AFDP problem-drinking count included 13 consequences and 12 dependence symptoms. The AHB problem-drinking count included 14 consequences and 13 dependence symptoms. See Table 1 for means and frequencies on the problem-drinking summary variables and Supplemental Appendix S2 for more detailed information about the items and their overlap between AFDP and AHB.

#### Marriage

Marital status items were used to classify participants at each wave into one of three mutually exclusive categories: “never married,” “became married” (first married since the preceding wave), and “post-marriage” (married at the preceding wave or prior to it). Of primary interest in this study was the contrast between the “never married” and “became married” groups, whereas the “post-marriage” group was created for data-analytic purposes (see Model-Building Stage 2 under Analytic Procedures).

#### Covariates

Sex (0 = male; 1 = female) and familial AUD (0 = negative; 1 = positive) were time-invariant binary variables.

## Analytic procedures

Analyses employed a structural equation modeling (SEM) framework for growth modeling (Muthén & Muthén, 1998–2017). Full information maximum likelihood estimation allowed for incomplete data. Robust standard errors accounted for AFDP’s sibling clustering in the AFDP models.

### Restructuring of wave-based data into age-binned data

Prior to growth modeling, the wave-based AFDP and AHB data were restructured into six discrete age bins including ages 17–20, 21–23, 24–26, 27–30, 31–34, and 35–39 (see Table 1 and Supplemental Appendix S1). This age binning is a common approach for addressing intrawave age variability in data from an accelerated longitudinal design (Prinzie & Onghena, 2005), and it thereby permitted straightforward tests of age moderation in our SEM-based growth models with time-varying marriage effects.^1^ Due to younger ages in the first current-study wave of AFDP relative to the first current-study wave of AHB, we retained the youngest age bin of 17–20 for AFDP but not AHB (see Table 1 and Supplemental Appendix S1). This decision resulted in exclusion of nine AHB person-observations for participants who were younger than age 21 at the first current-study wave of AHB. We also excluded one AFDP person-observation and four AHB person-observations with atypical age-by-wave patterns to prevent any participant from having multiple observations in a single age bin (e.g., an AHB participant assessed at age 31 at Wave 6 and age 34 at Wave 7 would otherwise have two person-observations that could be used for the 31–34 age bin).

### Model-building stage 1

**Figure 1:**
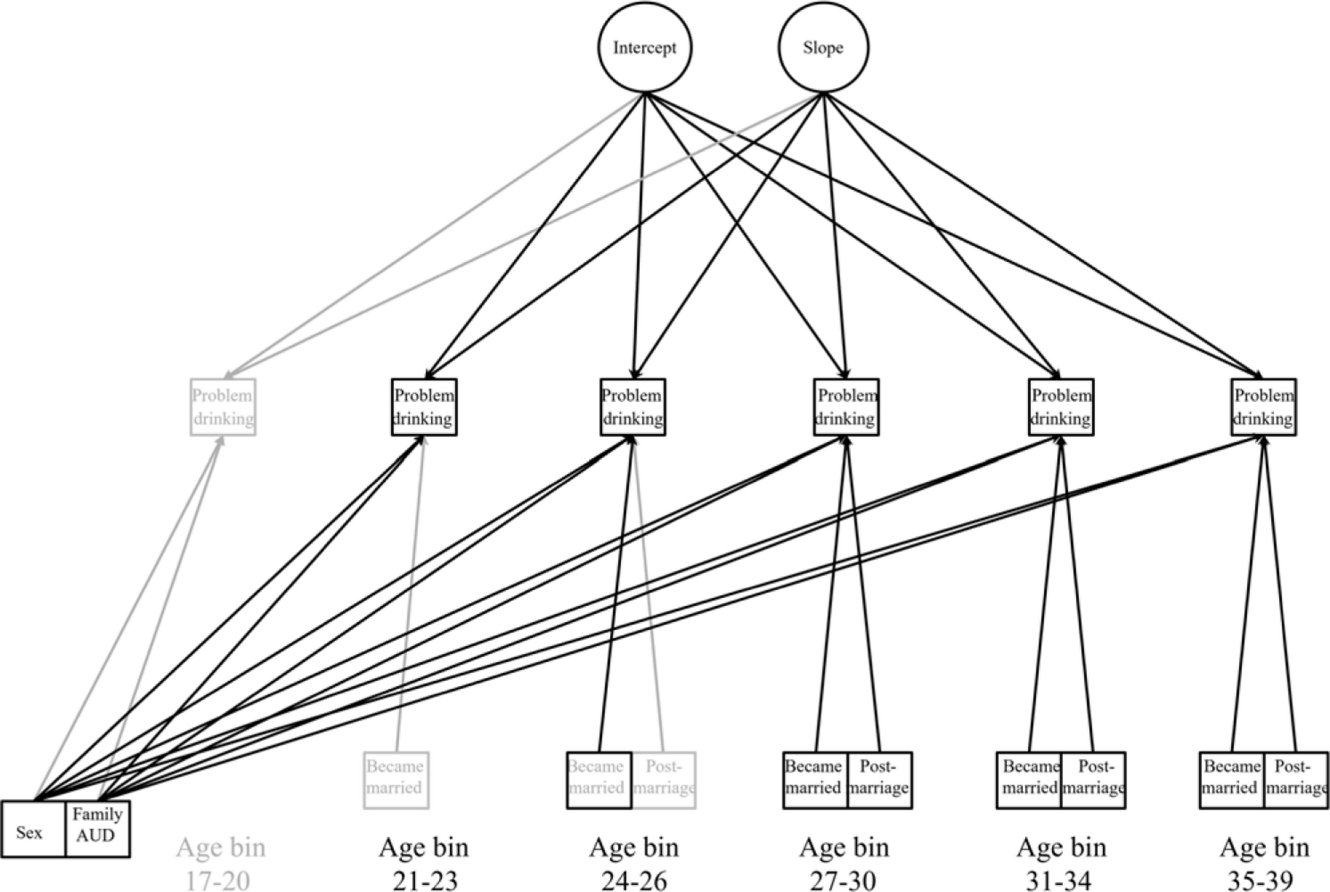
Path diagram of growth models testing age differences in the “became married” effect on problem-drinking change. Gray = excluded in AHB models (see Analytic Procedures section).

Multiple unconditional growth models of problem drinking were compared. In each model, the age bin-specific problem-drinking variables were modeled as indicators of problem-drinking change (see Figure 1). The different models that were contrasted made different distributional assumptions about the problem-drinking variables and specified different forms of problem-drinking change (see Table 2).

### Model-building stage 2

After selecting the best fitting unconditional growth model in model-building stage 1, model-building stage 2 involved estimating conditional growth models with predictors of problem drinking. This included time-varying marriage effects and time-invariant sex and familial AUD effects (see Figure 1).

For the marriage effects, at each age bin, the marriage variables were modeled as two dummy variables, one contrasting “never married” versus “became married” and one contrasting “never married” versus “post-marriage.” As noted in the Measures section, the contrast of primary interest was the first contrast of the “became married” versus “never married” groups. The second contrast involving the “post-marriage” group was primarily employed to facilitate homogeneity of the groups in the first contrast. An alternative approach of merely excluding those who could not be classified as either “never married” or “became married” could not be used in these analyses, given that members of the “post-marriage” group at one time point were often involved in the key contrast of “never married” versus “became married” at an earlier time point. Note that, as with other group comparisons using sets of dummy variables, the composition of the third “post-marriage” group (e.g., percent still married versus divorced) will not affect the contrast of the first and second groups. Note also that, by design, given our inclusion of only participants who were unmarried at the first time point (due to our primary interest in observing longitudinal transitions into marriage), “became married” effects could not be modeled at the first age bin, and “post-marriage” effects could not be modeled at the first three age bins. Although complex, this approach facilitates the key objective of allowing “became married” effects to be interpreted as prospective effects of becoming married as opposed to remaining never married, with this contrast predicting deviations in participants’ ongoing problem-drinking growth trajectories.

### Hypothesis tests of age differences in marriage effects

Two different types of Wald *χ*^2^ tests were conducted to test age differences in the “became-married effect.” An “omnibus” Wald *χ*^2^ test assessed whether model fit reduced significantly when constraining the became-married effect to equality across all age bins at which it was estimated. A “linear” Wald *χ*^2^ test provided a more specific and thus presumably more statistically powerful test of our age moderation hypothesis (Agresti, 2018). This test involved constraining the changes in the marriage effects from one wave to the next to be equivalent and then assessing if the resulting rate of linear change in the marriage effect differed significantly from zero.

## Results

### Results of model-building stage 1

In the first model-building stage comparing different distributional assumptions and different specified forms of problem-drinking change (see Table 2), the Bayesian information criterion (Schwartz, 1978) favored a linear negative-binomial count model for the AFDP data and a free-curve Poisson count model for the AHB data. Thus, these two models were retained and expanded upon in the second model-building stage.

Note that we also considered an alternative strategy of choosing a single modeling approach for both samples. Specifically, the model comparisons in Table 2 can be interpreted to indicate that a free-curve negative binomial model would be the most parsimonious model that could account for characteristics of both samples, accounting for the negative binomial dispersion in AFDP and the need for a freely estimated growth curve in AHB. In supplemental models that retained the free-curve negative binomial model for both AFDP and AHB in accordance with this alternative strategy (see Supplemental Appendix S3), resulting conclusions regarding our hypotheses were very similar to those from our primary analyses where different best-fitting models were retained for AFDP versus AHB.

### Results of model-building stage 2

As described below in greater detail, results generally supported our hypothesis by showing in both AFDP and AHB that the became-married effect generally decreased across ascending age bins and reached very small and nonsignificant magnitudes by the early-to-late 30s (see Table 3).

### AFDP-model hypothesis tests of age differences in marriage effects

**Figure 2:**
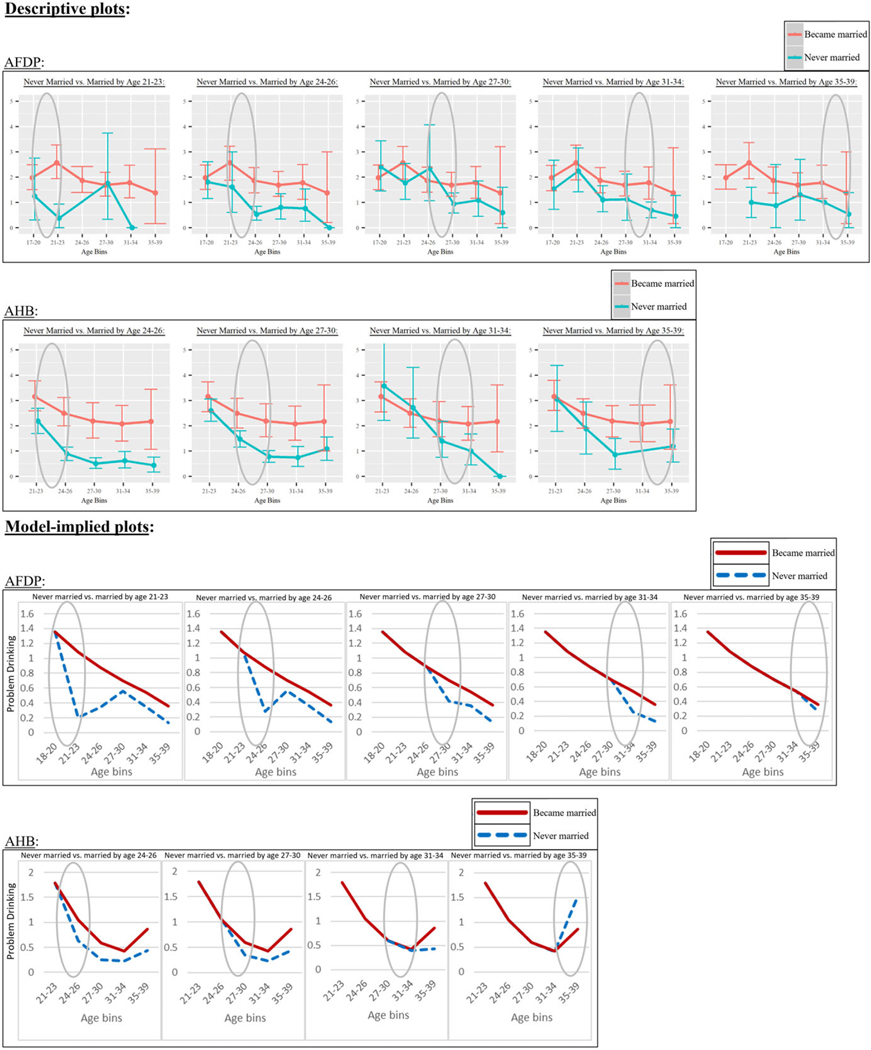
Descriptive plots (top panel) and model-implied plots (bottom panel) of age variability in the “became married” effect on problem-drinking reduction. The shaded circle in a given panel highlights parts of the lines that represent the age-specific “became married” effect of interest in that panel. For the descriptive plots, the lines connect group-specific means, with error bars around each mean. As noted in the main text, these plots support our conclusions from the model results and Wald *χ*^2^ test, as the plots also show trends across ascending ages where became-married effects (1) decreased in magnitude (see circled parts of descriptive and model-implied plots representing divergences of those who became married versus remained unmarried at different ages) and (2) decreased in statistical reliability (see error bars around means in the descriptive plots).

In the AFDP model (see Table 3), the *omnibus* Wald *χ*^2^ test did not show significant differences in became-married effects amongst the age bins (*χ*^2^(4)=5.899, *p* = .207). However, the *linear* Wald *χ*^2^ test did show a significant pattern of change in became-married effects across ascending age bins (*χ*^2^(1) = 4.169, *p* = .041). A more detailed characterization of how the AFDP marriage effects appeared to vary by age can be gleaned from model estimates of AFDP became-married effects at each age bin (see Table 3) and from descriptive and model-implied plots of AFDP became-married effects by age (see Figure 2). The AFDP model estimates showed a pattern in which the became-married effects decreased in magnitude somewhat uniformly across ascending age bins and (2) became nonsignificant (with an especially small magnitude) by the last age bin of 35–39. Further, the descriptive and model-implied plots also show trends where AFDP became-married effects decrease across ascending ages in both magnitude and statistical reliability (explained further in Figure 2 caption).

### AHB-model hypothesis tests of age differences in marriage effects

In the AHB model (see Table 3), significant effects were found for both the omnibus (*χ*^2^(3)=8.723, *p* = 0.033) and linear (*χ*^2^(1)= 4.774, *p* =.029) tests of differences in became-married effects across the age bins.

As with AFDP, the AHB model estimates also showed a pattern where the became-married effects decreased in magnitude somewhat uniformly across ascending age bins. Two differences from the AFDP results include that the AHB marriage effects (1) first reached nonsignificance at the second-to-last age bin of 31–34 (rather than at the last age bin in AFDP) and (2) appeared to reverse direction at the last age bin of 35–39 such that becoming married predicted increased problem drinking at these ages (albeit with only “marginal significance” of *p* = .067). As in AFDP, descriptive and model-implied plots also show trends where AHB became-married effects appear to decrease across ascending ages in both magnitude and statistical reliability (explained further in Figure 2 caption).

### Supplemental analyses

More complex analyses such as those formally testing moderation by sex and ethnicity were not feasible here due to sample size constraints (e.g., some such models were tested but failed to converge). Because sex is an especially important factor to consider in research on effects of family role transitions, we attempted to at least glean some preliminary insights into potential sex moderation by re-estimating our primary models separately in males and females (see Supplemental Appendix S4). These analyses yielded mixed conclusions. In AFDP, there did appear to be sex differences such that effects of becoming married did decrease by age among males in a way similar to our primary results, whereas effects of becoming married were significant across all ages among females. Further, the Wald tests of age reductions in marriage effects were significant for AFDP males (omnibus: *χ*^2^(4)=37.00 (*p* < .001); linear: *χ*^2^(1) = 11.67 (*p* < .001)) but not significant in AFDP females (omnibus: *χ*^2^(3) = 1.04 (*p* = .792); linear: *χ*^2^(1) = 0.03 (*p* = .888). In AHB, however, both males and females showed effects of becoming married that decreased by age. The Wald tests of age reductions in marriage effects only reached or approached significance for AHB males (omnibus: *χ*^2^(3) = 7.55 (*p* = .056); linear: *χ*^2^(1) = 3.91 (*p* = .048)), but the nonsignificant of these Wald tests among AHB females (omnibus: *χ*^2^(3) = 3.01 (*p* = .390); linear: *χ*^2^(1) = 1.07 (*p* = .301)) should be interpreted with caution due to the relative statistical-power limitation of these models.

Supplemental analyses were also conducted to investigate confounding effects of marital recency as a potential threat to internal validity and thus as a potential alternative explanation for our apparent evidence for age-related reductions in marriage effects. In Supplemental Appendix S4, descriptive analyses do show a pattern of greater recency of marriage among younger “became married” participants. This could tentatively support an argument that weaker marriage effects were observed at later ages because those later marriages were less recent in relation to the time point when problem-drinking change was subsequently assessed. However, in supplemental models that parsed “became married” groups into high-recency and low-recency subgroups at each age bin, there was little evidence for stronger effects of more recent marriages (see Supplemental Appendix S5). In fact, across the age bins, it often appeared that less recency was associated with a slightly stronger “became married” effect. This is opposite to the direction of recency effects that would be required for a confound of age with recency to be a plausible alternative explanation for our findings. Thus, we are confident these supplemental analyses effectively ruled out this potential threat to internal validity and thereby provided additional support for our evidence of greater marriage effects at younger ages.

## Discussion

This study investigated age differences in marriage effects on problem-drinking reduction, a topic that aligns with a broader empirical objective of understanding differences across the adult life span in factors that influence problem drinking and problem-drinking desistance. As hypothesized, the marriage effect was strongest in early young adulthood and decreased thereafter with age, reaching very small (and nonsignificant) magnitudes by the 30s. Importantly, this finding was replicated in parallel analyses with two independent samples (AFDP and AHB; see Method section).

### Conceptual interpretations of findings

As reviewed in the Introduction section, reasons to predict that family roles like marriage may exert particularly strong beneficial effects earlier in adulthood include (1) the importance of such adult role transitions during young adulthood per established developmental theory (e.g., Erikson, 1968) and (2) broader arguments that contextual effects may diminish with age across adulthood as individuals increasingly construct their own environments (Kendler et al., 2007; Scarr & McCartney, 1983). In light of these ideas, our current results may more broadly indicate a peak in earlyto-middle young adulthood in individuals’ tendency and/or capacity for adapting to new contextual demands. However, this broader potential interpretation is indeed quite speculative.

As noted earlier, effects of family roles like marriage are often interpreted through the lens of sociologic role incompatibility theory (Yamaguchi & Kandel, 1985). Thus, our theoretical basis for making our hypotheses and interpreting our results largely reflects concepts from role incompatibility theory and our conceptual extensions of these concepts into a life span developmental framework. From this view, our results may reflect the fact that earlier marriages more often prompt behavior change such as drinking reductions because of a need to adapt to the marital role and thereby resolve conflict between behaviors and the role’s demands (i.e., role socialization as a means of resolving role incompatibility). In contrast, those at later ages of adulthood may more often preemptively avoid such conflict by avoiding marriage when it can be expected to conflict with established behaviors like their drinking (i.e., role selection as a means of avoiding role incompatibility). In addition to role selection via role avoidance, older individuals may also engage in more role selection via choosing marital partners with characteristics that will decrease the likelihood/extent of role incompatibility. Thus, problem drinkers who marry at later ages may more often marry heavy-drinking or drinking-permissive spouses. Also, older individuals may engage in more selection via role departure, perhaps with marriage-related role incompatibility more often resulting in divorce rather than behavioral changes like adaptive drinking reductions. Future research should investigate each of these three specific forms of role selection to assess if each increasingly occurs with age and thereby contributes to the age reductions in marriage effects shown in our results.

There are other plausible potential mechanisms of marriage’s beneficial effects aside from those related to role incompatibility, and these represent other conceptual interpretations that could further inform an understanding of our findings. These other potential mechanisms include marriage-driven personality maturation (e.g., increased conscientiousness; Lee et al., 2015; Littlefield et al., 2009) and marriage-driven relationship bonding (e.g., Roberts & Chapman, 2000; Sampson et al., 2006), both of which could reduce with age to produce the currently observed age reductions in marriage effects on drinking change. Further, other viable interpretations of our findings could emphasize potential life span developmental differences in the typical nature of marriage itself. For instance, later marriages may be less conventional with regard to the individuals in the marriage and/or individually varying characteristics of the marital role (Uecker & Stokes, 2008), thus perhaps bringing fewer marital-role demands of the sort that prompt drinking-related reductions. Yet other viable interpretations could emphasize life span developmental differences in the landscape of other drinking-related risk factors. For instance, due to a greater prevalence of various contextual (e.g., peerrelated) risk factors at younger ages (Schulenberg et al., 2018), earlier marriages may more often serve to buffer against these contextual risks that could otherwise prompt problem-drinking continuation or escalation. Thus, multiple nonmutually exclusive mechanisms might explain the currently observed age-related decline in the magnitude of the marriage effect on problem-drinking reductions.

### The importance of role timing

Regardless of these alternative interpretations, our findings highlight that the timing of family role transitions like marriage deserves empirical attention. Other research has characterized how certain risk factors can delay timing of marriage and parent-hood (e.g., Waldron et al., 2011), and our findings exemplify how such delays could diminish the benefits these family role transitions convey. Our findings also complement work (e.g., Little et al., 2009) suggesting limited salutary effects of non-normatively early transitions to family roles (e.g., in late adolescence). Thus, there may be nonlinear moderation by age such that marriage’s beneficial effects peak around early-to-middle young adulthood, and future research should investigate the extent that the same or different constructs explain marriage’s weaker effects prior to and after these ages (i.e., “mediated moderation” testing more conceptual moderators that may mediate age’s moderating effects; MacKinnon & Fairchild, 2009).

Regarding public health implications, given recent demographic shifts toward later marriage and parenthood (Furstenberg, 2010), our findings suggest that such shifts could reduce the magnitude of family role-driven drinking reductions in the population. Indeed, according to US epidemiologic data (Schulenberg et al., 2019), more recent cohorts have shown a relatively blunted pattern of drinking reductions during the 20s (e.g., Jager et al., 2015; Patrick et al., 2019). Given our current evidence for age specificity of marriage effects, it is sensible to speculate that this trend toward less dramatic young adult “maturing out” in recent years may have been influenced by the trends toward later marriage and parenthood. However, this raises questions about generalizability of our findings to more recent cohorts. Thus, a key next step in this line of research should involve cross-cohort longitudinal analyses assessing if there have been shifts across recent cohorts in the ages at which family role effects are strongest.

### Clinical implications

Our findings reflect one specific instantiation of the ways that life span developmental research can help guide interventions by better accounting for variability in key risk and protective processes across different developmental periods of adulthood. Our findings suggest that young–adult interventions should emphasize processes of adaptation to adult roles like marriage. Such interventions may be able to jumpstart and amplify role adaptation processes already primed to occur naturally in this developmental period, which may make this strategy particularly impactful and efficient. Importantly, this clinical strategy could also include efforts to spur *anticipatory* role adaptation, consistent with evidence that anticipatory drinking reductions prior to marriage can occur naturally (Bachman et al., 2002). The above arguments are not meant to imply that midlife problem drinkers could not also benefit from interventions emphasizing role adaptation. However, it may at least be useful for clinicians to anticipate that, when using this strategy at older ages, clinicians may (1) encounter more potential barriers and (2) more often be aiming to initiate role adaptation processes rather than amplifying naturally ongoing ones.

### The broader objective of understanding life span variability in mechanisms of problem-drinking desistance

There is a dearth of research on developmental differences across adulthood in patterns and predictors of problem-drinking reduction, despite the fact that key unique insights have been gleaned from developmental analyses focused on heterogeneity across earlier developmental periods (e.g., from childhood to late adolescence; Chassin et al., 2013; NIAAA, 2017). The current study indicates that marriage may be a particularly important mechanism of problem-drinking reduction in young adulthood relative to later periods of the adult life span. However, given the ongoing increases in older adult problem drinking that coincide with aging of the “baby boomer” generation (Han et al., 2009), future research should continue to examine other possible mechanisms that may be important in later periods of adulthood.

### Limitations

Despite its strengths, there are noteworthy limitations of the current study. First, our samples were assessed from the late teens to late 30s, and future research spanning the entire adult life span is needed. Second, although a confound should be noted between age of marriage and marital recency (i.e., years since marriage at a given longitudinal observation), supplemental analyses with the AFDP sample ruled out marital recency as an alternative explanation for our evidence of stronger marriage effects at younger ages (see Supplemental Appendix S4). Third, AFDP and AHB had notable sample differences, which created some differences in the analyses (e.g., AHB’s more restricted lower bound of age required exclusion of the 17–20-year-old age bin from AHB analyses). Although such differences somewhat limit comparability across studies, they also highlight the generalizability reflected by our replication of findings in both studies. Despite replication, these samples include less recent cohorts of high-risk participants that are nonrepresentative of other populations, so the current analyses should be replicated in other samples. Note though that the AFDP and AHB studies took place over similar time periods (from around the mid-80s to the early 2000s), so there is unlikely to be cause for concern regarding cohort differences between the two studies that could jeopardize their comparability.

Fourth, this study also did not attend to other romantic-relationship transitions (e.g., cohabitation, divorce, remarriage), other life course events (e.g., parenthood, education/employment transitions), moderation by sex, sexual minority status, ethnicity, culture, nationality, familial AUD, or other comorbid mental health conditions. For the interested reader, Supplemental Appendices S6 and S7 (1) characterize ways that life course events other than marriage vary in prevalence across this study’s age bins and (2) present supplemental models supporting the robustness of findings while accounting for potential confounding effects of parenthood and marital engagement. Unfortunately, the present study lacks available data to clarify whether courtship or cohabitation effects may vary by age and how this may confound our results. However, in contrast to marriage, past research has not tended to link courtship or cohabitation to drinking reductions (Caetano et al., 2006; Fryar et al., 2007; Joung et al., 1995; Power et al., 1999; Schoenborn, 2004), so we believe it is unlikely that our findings are spuriously driven by confounding age differences in cohabitation effects.

## Conclusions

Parallel analyses in two high-risk samples confirmed our prediction that the effect of marriage on problem-drinking reductions would be strongest in young adulthood and decrease thereafter with age. This novel evidence for the importance of family role timing as a moderator of family role effects has important public health and clinical implications. More broadly, findings highlight the importance of a life span developmental approach for understanding problem-drinking desistance and guiding life span developmentally informed intervention strategies.

## Supplementary Material

1

## Figures and Tables

**Table 1.** Characteristics of the current-study AFDP and AHB samples, both in original wave-based structure and the current study’s age-binned structure

AFDP (*N* = 577)										
	Wave 4 (ages 17–26)	Wave 5 (ages 21–33)	Wave 6 (ages 27–39)	-	Age bin 17–20	Age bin 21–23	Age bin 24–26	Age bin 27–30	Age bin 31–34	Age bin 35–39
*N*	527	516	507	-	324	250	330	300	281	65
Age mean (*SD*)	20.6 (2.0)	25.9 (2.2)	32.2 (2.4)	-	19.3 (1.0)	22.5 (1.0)	25.3 (0.8)	29.2 (1.1)	32.7 (1.1)	36.8 (1.3)
% Became married (*n*)	0% (*n* = 0)	31% (*n* = 161)	29% (*n* = 145)	-	0% (*n* = 0)	6% (*n* = 16)	25% (*n* = 84)	35% (*n* = 104)	32% (*n* = 89)	20% (*n* = 13)
% Post-marriage (*n*)	0% (*n* = 0)	0% (*n* = 0)	29% (*n* = 148)	-	0% (*n* = 0)	0% (*n* = 0)	0% (*n* = 0)	13% (*n* = 38)	29% (*n* = 82)	43% (*n* = 28)
Problem-drinking mean (*SD*)	1.9 (3.2)	1.4 (2.6)	1.3 (2.7)		1.9 (3.2)	2.1 (3.1)	1.4 (2.8)	1.3 (2.5)	1.2 (2.7)	0.8 (2.6)
% Problem drinking=0	262 (49.7%)	305 (59.1%)	332 (65.5%)		167 (51.5%)	112 (44.8%)	196 (59.4%)	182 (60.7%)	191 (68.0%)	51 (78.5%)
% Problem drinking=1	85 (16.1%)	74 (14.3%)	62 (12.2%)		49 (15.1%)	47 (18.8%)	49 (14.8%)	35 (11.7%)	34 (12.1%)	7 (10.8%)
% Problem drinking≥2	180 (34.1%)	137 (26.7%)	113 (22.3%)		108 (33.0%)	91 (36.4%)	85 (25.6%)	83 (27.5)	56 (22.2%)	7 (10.5)
AHB (*N* = 441)										
	Wave 4 (ages 20–30)	Wave 5 (ages 23–33)	Wave 6 (ages 28–36)	Wave 7 (ages 33–37)	-	Age bin 21–23	Age bin 24–26	Age bin 27–30	Age bin 31–34	Age bin 35–39
*N*	432	423	380	355	-	426	419	378	244	123
Age mean (*SD*)	21.3 (0.9)	24.5 (1.0)	29.0 (0.8)	34.3 (0.7)	-	21.2 (0.5)	24.4 (0.5)	28.9 (0.7)	33.8 (0.5)	35.2 (0.5)
% Became married (*n*)	0% (*n* = 0)	30% (*n* = 127)	35% (*n* = 134)	12% (*n* = 44)	-	0% (*n* = 0)	30% (*n* = 127)	35% (*n* = 131)	12% (*n* = 29)	15% (*n* = 18)
% Post-marriage (*n*)	0% (*n* = 0)	0% (*n* = 0)	32% (*n* = 122)	79% (*n* = 279)	-	0% (*n* = 0)	0% (*n* = 0)	32% (*n* = 122)	76% (*n* = 186)	76% (*n* = 93)
Problem-drinking mean (*SD*)	2.7 (3.1)	1.7 (2.5)	1.0 (2.0)	1.0 (1.9)	-	2.7 (3.1)	1.7 (2.5)	1.0 (1.9)	1.0 (2.0)	1.2 (2.1)
% Problem drinking=0	110 (26.4%)	190 (43.9%)	241 (62%)	233 (64.6%)		113 (26.2%)	188 (44.1%)	241 (62.5)	183 (65.8%)	74 (58.6%)
% Problem drinking=1	85 (19.5%)	89 (21.4%)	56 (15.3%)	44 (12.8%)		82 (19.3%)	89 (21.5%)	56 (15.4%)	28 (11.8%)	17 (14.4%)
% Problem drinking≥2	237 (54.1%)	144 (34.7)	83 (22.7%)	78 (22.6%)		231 (54.5%)	142 (34.4%)	81 (22.1%)	33 (22.4%)	32 (27%)

*Note*. See Supplemental Appendix S1 for more detailed information regarding age and the problem-drinking outcome variable in AFDP and AHB.

**Table 2.** BIC comparisons of different unconditional growth models

	AFDP	AHB
MLR		
Linear	7536.279	6763.213
Quadratic	7540.56^1^	6701.719
Free curve	7526.758	6704.178
Poisson		
Linear	4983.012	4941.773
Quadratic	4893.469	4912.118
Free curve	5036.413	4911.52
NB		
Linear	4786.812	4945.445
Quadratic	4811.812	4931.881
Free curve	4802.48	4914.59
ZIP		
Linear	4832.882	4945.738
Quadratic	4874.701	4983.043
Free curve	4892.184	4931.805

*Note*. BIC = Bayesian information criterion; MLR = maximum likelihood estimation with robust standard errors; Poisson = Poisson count modeling; NB = negative-binomial count modeling; ZIP = zero-inflated Poisson count modeling. Linear = an intercept factor and a linear-slope factor; Quadratic = an intercept factor and linear- and quadratic-slope factors; Free curve = an intercept factor and a freely estimated slope factor.

1 This model constrained the quadratic-slope factor variance to zero because an initial model estimating the quadratic-slope factor variance was nonconvergent.

**Table 3.** Results of ADFP and AHB growth models testing age variability in effects of marriage on problem-drinking reductions

	AFDP model (linear NB)			AHB model (free-curve Poisson)		
	Estimates	*p* Value	95% CIs	Estimates	*p* Value	95% CIs
Effects on age 17–20 problem drinking						

Sex (0 = male; 1 = female)	−0.832	.000	−1.225, −0.438	-	-	-

Familial AUD (0 = negative; 1 = positive)	0.425	.036	0.029, 0.821	-	-	-

Effects on age 21–23 problem drinking						

**Became married by age 21–23**	**−1.393**	**.004**	**−2.335, −0.452**	-	-	-

Sex (0 = male; 1 = female)	−0.686	.000	−0.979, −0.393	−0.680	.000	−0.887, −0.473

Familial AUD (0 = negative; 1 = positive)	0.461	.001	0.198, 0.724	0.370	.002	0.141, 0.600

Effects on age 24–26 problem drinking						

**Became married by age 24–26**	**−0.927**	**.000**	**−1.318, −0.535**	**−0.414**	**.002**	**−0.672, −0.156**

Sex (0 = male; 1 = female)	−0.231	.240	−0.617, 0.155	−0.614	.000	−0.836, −0.391

Familial AUD (0 = negative; 1 = positive)	0.181	.286	−0.152, 0.514	0.307	.007	0.084, 0.600

Effects on age 27–30 problem drinking						

**Became married by age 27–30**	**−0.350**	**.047**	**−0.696, −0.004**	**−0.361**	**.001**	**−0.580, −0.142**

Post-marriage by age 27–30	−0.166	.553	−0.714, 0.382	−0.537	.000	−0.817, −0.258

Sex (0 = male; 1 = female)	−0.556	.001	−0.887, −0.224	−0.434	.001	−0.682, −0.186

Familial AUD (0 = negative; 1 = positive)	0.376	.011	0.088, 0.664	0.249	.050	0.000, 0.498

Effects on age 31–34 problem drinking						

**Became married by age 313–4**	**−0.424**	**.015**	**−0.765, −0.084**	**−0.015**	**.928**	**−0.352, 0.321**

Post-marriage by age 313–4	−0.248	.154	−0.590, 0.093	−0.348	.006	−0.596, −0.100

Sex (0 = male; 1 = female)	−0.462	.007	−0.796, −0.127	−0.471	.000	−0.735, −0.207

Familial AUD (0 = negative; 1 = positive)	0.276	.130	−0.081, 0.632	0.353	.012	0.079, 0.627

Effects on age 353–9 problem drinking						

**Became married by age 353–9**	**−0.163**	**.747**	**−1.156, 0.830**	**0.505**	**.067**	**−0.035, 1.045**

Post-marriage by age 353–9	−0.490	.187	−1.218, 0.238	−0.613	.184	−1.516, 0.291

Sex (0 = male; 1 = female)	−0.075	.848	−0.842, 0.693	−0.195	.459	−0.713, 0.322

Familial AUD (0 = negative; 1 = positive)	−0.052	.899	−0.852, 0.749	0.017	.955	−0.580, 0.615

**Wald *χ*^2^ *omnibus* test of age moderation of the “became married” effect**	***χ*^2^(4) = 5.89 (*p* = .207)**			***χ*^2^(3) = 8.72 (*p* = .033)**		

**Wald *χ*^2^ *linear* test of age moderation of the “became married” effect**	***χ*^2^ (1)= 4.16 (*p* = .041)**			***χ*^2^(1) = 4.77 (*p* = .029)**		

Growth factor means						

Intercept factor	0.398	.027	-	0.744	.000	-

Slope factor	−1.440	.014	-	−0.515	.000	-

Growth factor covariance						

Intercept factor with slope factor	0.561	.481	-	0.101	.025	-

Intercept factor loadings						

Age 17–20 problem drinking	@1	-	-	-	-	-

Age 21–23 problem drinking	@1	-	-	@1	-	-

Age 24–26 problem drinking	@1	-	-	@1	-	-

Age 27–30 problem drinking	@1	-	-	@1	-	-

Age 31–34 problem drinking	@1	-	-	@1	-	-

Age 35–39 problem drinking	@1	-	-	@1	-	-

Slope factor loadings						

Age 17–20 problem drinking	@0	-	-	-	-	-

Age 21–23 problem drinking	@0.17	-	-	@0	-	-

Age 24–26 problem drinking	@0.33	-	-	@1	-	-

Age 27–30 problem drinking	@0.50	-	-	2.151	0.000	-

Age 31–34 problem drinking	@0.69	-	-	2.823	0.000	-

Age 35–39 problem drinking	@1	-	-	1.342	0.365	-

*Note*. Reference group: never married. Standardized estimates from the Mplus “STDY” standardized solution are reported (Muthén & Muthén, 1998–2017). Poisson = Poisson count modeling; NB = negative-binomial count modeling. Linear = an intercept factor and a linear-slope factor; free curve = an intercept factor and a freely estimated slope factor. The “@” symbol indicates that a parameter was constrained to the given value. Bolding is intended to bring readers attention to the model estimates that are of primary interest to the study.

## References

[R1] Agresti A. (2018). An introduction to categorical data analysis. John Wiley & Sons.

[R2] American Psychiatric Association. (1980). Diagnostic and statistical manual of mental disorders (3rd ed.)

[R3] American Psychiatric Association. (2013). Diagnostic and statistical manual of mental disorders: DSM-5 (5th ed.).

[R4] Bachman JG, O’Malley PM, Schulenberg JE, Johnston LD, Bryant AL, & Merline AC (2002). The decline of substance use in young adulthood: Changes in social activities, roles, and beliefs. Lawrence Erlbaum Associates. 10.4324/9781410606013

[R5] Bennett M, Mccrady B, Johnson V, & Pandina R. (1999). Problem drinking from young adulthood to adulthood: Patterns, predictors and outcomes. Journal of Studies on Alcohol, 60(5), 605–614. 10.15288/jsa.1999.60.605 10487729

[R6] Benson JE& Elder GH (2011). Young adult identities and their pathways: A developmental and life course model. Developmental Psychology, 47(6), 1646–1657. 10.1037/a0023833 21668096 PMC3792649

[R7] Caetano R, Ramisetty-Mikler S, Floyd LR, & McGrath C. (2006). The epidemiology of drinking among women of child-bearing age. Alcoholism, Clinical and Experimental Research, 30(6), 1023–1030. 10.1111/j.1530-0277.2006.00116.x 16737461

[R8] Chassin L, Barrera M, Bech K, & Kossak-Fuller J. (1992). Recruiting a community sample of adolescent children of alcoholics: A comparison of three subject sources. Journal of Studies on Alcohol, 53, 316–319. 10.15288/jsa.1992.53.316 1619925

[R9] Chassin L, Rogosch F, & Barrera M. (1991). Substance use and symptomatology among adolescent children of alcoholics. Journal of Abnormal Psychology, 100(4), 449–463. 10.1037/0021-843X.100.4.449 1757658

[R10] Chassin L, Sher KJ, Hussong A, & Curran P. (2013). The developmental psychopathology of alcohol use and alcohol disorders: Research achievements and future directions. Development and Psychopathology, 25, 1567–1584. 10.1017/S0954579413000771 24342856 PMC4080810

[R11] Crews T, & Sher KJ (1992). Using adapted Short MASTs for assessing parental alcoholism: Reliability and validity. Alcoholism: Clinical and Experimental Research, 16, 427–448. 10.1111/j.1530-0277.1992.tb01420.x 1626659

[R12] Curran PJ, McGinley JS, Bauer DJ, Hussong AM, Burns A, Chassin L, Sher KJ, & Zucker R. (2014). A moderated nonlinear factor model for the development of commensurate measures in integrative data analysis. Multivariate Behavioral Research, 49(3), 214–231. 10.1080/00273171.2014.889594 25960575 PMC4423418

[R13] Edwards G, & Gross MM (1976). Alcohol dependence: Provisional description of a clinical syndrome. British Medical Journal, 1, 1058–1061. 773501 10.1136/bmj.1.6017.1058 PMC1639901

[R14] Endicott J, Andreasen N, & Spitzer RL (1978). Family History-Research Diagnostic Criteria (FH-RDC). New York State Psychiatric Institute.

[R15] Erikson EH (1968). Identity: youth and crisis. Norton & Co.

[R16] Fryar CD, Hirsch R, Porter KS, Kottiri B, Brody DJ, & Louis T. (2007). Drug use and sexual behaviors reported by adults: United States, 1999–2002. Advance Data, (384), 1–14. 17668724

[R17] Furstenberg FF Jr (2010). On a new schedule: Transitions to adulthood and family change. The Future of Children, 20, 67–87. 10.1353/foc.0.0038 20364622

[R18] Giordano PC, Stephen A, Cernkovich SA, & Rudolph JL (2002). Gender, crime, and desistance: Toward a theory of cognitive transformation. American Journal of Sociology, 107, 990–1064. 10.1086/343191

[R19] Grant BF, Kaplan KK, Stinson FS (2005). Source and accuracy statement for the wave 2 national epidemiologic survey on alcohol and related conditions. National Institute on Alcohol Abuse and Alcoholism.

[R20] Grimm K, Ram N, & Estabrook R. (2017). Growth modeling: structural equation and multilevel modeling approaches (methodology in the social sciences). Guilford Press.

[R21] Han B, Gfroerer JC, Colliver JD, & Penne MA (2009). Substance use disorder among older adults in the United States in 2020. Addiction, 104, 88–96. 10.1111/j.1360-0443.2008.02411.x 19133892

[R22] Hussong AM, Curran PJ, & Bauer DJ (2013). Integrative data analysis in clinical psychology research. Annual Review of Clinical Psychology, 9, 61–89. 10.1146/annurev-clinpsy-050212-185522 PMC3924786 23394226

[R23] Jager J, Keyes KM, & Schulenberg JE (2015). Historical variation in young adult binge drinking trajectories and its link to historical variation in social roles and minimum legal drinking age. Developmental Psychology, 51(7), 962–974. 10.1037/dev0000022 26010381 PMC4517691

[R24] Joung IM, Stronks K, van de Mheen H, & Mackenbach JP (1995). Health behaviours explain part of the differences in self reported health associated with partner/marital status in The Netherlands. Journal of Epidemiology and Community Health, 49(5), 482–488. 10.1136/jech.49.5.482 7499990 PMC1060151

[R25] Kendler KS, Jacobson KC, Gardner CO, Gillespie N, Aggen SA, & Prescott CA (2007). Creating a social world: a developmental twin study of peer-group deviance. Archives of General Psychiatry, 64, 958. 10.1001/archpsyc.64.8.958 17679640 PMC4246499

[R26] Lee MR, Boness CL, McDowell YE, Vergés A, Steinley DL, & Sher KJ (2018). Desistance and severity of alcohol use disorder: A lifespan-developmental investigation. Clinical Psychological Science, 6, 90–105. 10.1177/2167702617736852 29335673 PMC5766269

[R27] Lee MR, Chassin L, & MacKinnon D. (2015). Role transitions and young adult maturing out of heavy drinking: Evidence for larger effects of marriage among more severe pre-marriage problem drinkers. Alcoholism: Clinical and Experimental Research, 39, 1064–1074. 10.1111/acer.12715 26009967 PMC4452406

[R28] Lee MR, Ellingson JM, & Sher KJ (2015). Integrating social-contextual and intrapersonal mechanisms of “maturing out”: Joint influences of familial role transitions and personality maturation on problem drinking reductions. Alcoholism: Clinical and Experimental Research, 39(9), 1775–1787. 10.1111/acer.12816 26247314 PMC4558380

[R29] Lee MR & Sher KJ (2018). “Maturing out” of binge and problem drinking. Alcohol Research: Current Reviews, 39(1), 31–42. 30557146 10.35946/arcr.v39.1.06 PMC6104962

[R30] Lee MR, Zhao Y, Britton T, Saviano J, Kideys K, Li Y, Boulter C, Frick E, & Sher KJ (in press) Lifespan developmental perspectives on natural mechanisms of cessation of risky alcohol use and recovery from alcohol use disorder. In Witkiewitz K, & Tucker JA (Eds.), Pathways to recovery from alcohol use disorder. Cambridge University Press.

[R31] Little M, Handley E, Leuthe E, & Chassin L. (2009). The impact of parenthood on alcohol consumption trajectories: Variations as a function of timing of parenthood, familial alcoholism, and gender. Development and psychopathology, 21, 661–682. 10.1017/S0954579409000352 19338703 PMC2680497

[R32] Littlefield AK, Sher KJ, & Wood PK (2009). Is ‘maturing out’ of problematic alcohol involvement related to personality change? Journal of Abnormal Psychology, 118(2), 360–374. 10.1037/a0015125 19413410 PMC2742487

[R33] MacKinnon DP, & Fairchild AJ (2009). Current directions in mediation analysis. Current Directions in Psychological Science, 18(1), 16. 10.1111/j.1467-8721.2009.01598.x 20157637 PMC2821103

[R34] Miller WR, & Rollnick S. (2002). Motivational interviewing: Preparing people for change (2nd ed). Guilford Press.

[R35] Muthén LK and Muthén BO (1998–2017). Mplus user’s guide (8th ed.) Muthén & Muthén

[R36] National Institute on Alcohol Abuse and Alcoholism. (2017). The National Institute on Alcohol Abuse and Alcoholism Strategic Plan 2017–2021. Retrieved April 19, 2021, from https://www.niaaa.nih.gov/sites/default/files/StrategicPlan_NIAAA_optimized_2017-2020.pdf

[R37] National Institute on Alcohol Abuse and Alcoholism. (2020). Alcohol Facts and Statistics. Retrieved April 19, 2021, from https://www.niaaa.nih.gov/sites/default/files/AlcoholFactsAndStats.pdf

[R38] Neve RJM, Lemmens PH, & Drop MJ (2000). Changes in alcohol use and drinking problems in relation to role transitions in different stages of the life course. Substance Abuse, 21(3), 163–178. 10.1080/08897070009511430 12466657

[R39] O’Malley PM (2004). Maturing out of problematic alcohol use. Alcohol Research and Health, 28, 202–204.

[R40] Patrick ME, Terry-McElrath YM, Lanza ST, Jager J, Schulenberg JE, & O’Malley PM (2019). Shifting age of peak binge drinking prevalence: Historical changes in normative trajectories among young adults aged 18 to 30. Alcoholism, Clinical and Experimental Research, 43(2), 287–298. 10.1111/acer.13933 30645773 PMC6432634

[R41] Power C, Rodgers B, & Hope S. (1999). Heavy alcohol consumption and marital status: Disentangling the relationship in a national study of young adults. Addiction (Abingdon, England), 94(10), 1477–1487. 10.1046/j.1360-0443.1999.941014774.x 10790900

[R42] Prescott C, & Kendler K. (2001). Associations between marital status and alcohol consumption in a longitudinal study of female twins. Journal of Studies on Alcohol, 62(5), 589–604. 10.15288/jsa.2001.62.589 11702798

[R43] Prinzie P, & Onghena P. (2005). Cohort sequential design. In Everitt BS & Howell D. (Eds.), Encyclopedia of statistics in behavioral science (vol. 1, pp.319–322). John Wiley & Sons, Ltd.

[R44] Roberts BW, & Chapman CN (2000). Change in dispositional well-being and its relation to role quality: A 30-year longitudinal study. Journal of Research in Personality, 34(1), 26–41. 10.1006/jrpe.1999.2259

[R45] Robins LN, Helzer JE, Ratcliff KS, & Seyfried W (1982). Validity of the Diagnostic Interview Schedule, Version II: DSM-III diagnoses. Psychological Medicine, 12, 855–870. 10.1017/S0033291700049151 7156256

[R46] Robins LN, Helzer JE, Croughan JL, & Ratcliff KS (1981). National institute of mental health diagnostic interview schedule: Its history, characteristics, and validity. Archives of General Psychiatry, 38, 381–389. 10.1001/archpsyc.1981.01780290015001 6260053

[R47] Sampson RJ, Laub JH, & Wimer C. (2006). Does marriage reduce crime? A counterfactual approach to within-individual causal effects. Criminology, 44(3), 465–508. 10.1111/j.1745-9125.2006.00055.x

[R48] Scarr S, & McCartney K. (1983). How people make their own environments: A theory of genotype → environment effects. Child Development, 424–435. 10.1111/j.1467-8624.1983.tb03884.x 6683622

[R49] Schoenborn CA (2004). Marital status and health: United States, 1999–2002 Advance data from vital and health statistics; no. 351. National Center for Health Statistics. 15633583

[R50] Schulenberg J, Maslowsky J, & Jager J. (2018). Substance use and abuse during adolescence and the transition to adulthood are developmental phenomena: Conceptual and empirical considerations. In Fitzgerald HE, & Puttler LI (Eds.), Alcohol use disorders: A developmental science approach to etiology. Oxford University Press. 10.1093/oso/9780190676001.003.0012

[R51] Schulenberg JE, Johnston LD, O’Malley PM, Bachman JG, Miech RA & Patrick ME (2019). Monitoring the Future national survey results on drug use, 1975–2018: Volume II, College students and adults ages 19–60. Institute for Social Research, The University of Michigan. Retrieved April 19, 2021, from http://monitoringthefuture.org/pubs.html#monographs

[R52] Schwartz G. (1978). Estimating the dimension of a model. Annals of Statistics, 6, 461–464. 10.1214/aos/1176344136

[R53] Selzer M. (1971). The Michigan Alcoholism Screening Test: The quest for a new diagnostic instrument. American Journal of Psychiatry, 172, 1653–1658. 10.1176/ajp.127.12.1653 5565851

[R54] Selzer M, Vinokur A, & van Rooijen L. (1975). A self-administered Short Michigan Alcoholism Screening Test (SMAST). Journal of Studies on Alcohol, 36, 117–126. 10.15288/jsa.1975.36.117 238068

[R55] Sher KJ, & Vergés A. (2016). Introduction and overview. In Sher KJ (Ed.), Oxford handbook of substance use and substance use disorders (pp. 449–482). Oxford University Press.

[R56] Sher KJ, Walitzer KS, Wood PK, & Brent EE (1991). Characteristics of children of alcoholics: Putative risk factors, substance use and abuse, and psychopathology. Journal of Abnormal Psychology, 100, 427–448. 10.1037/0021-843X.100.4.427 1757657

[R57] Temple M, Fillmore K, Hartka E, Johnstone B, Leino E, & Motoyoshi M. (1991). A meta-analysis of change in marital and employment status as predictors of alcohol consumption on a typical occasion. British Journal of Addiction, 86(10), 1269–1281. 10.1111/j.1360-0443.1991.tb01703.x 1836409

[R58] Uecker JE, & Stokes CE (2008). Early marriage in the United States. Journal of Marriage and Family, 70(4), 835–846. 10.1111/j.1741-3737.2008.00530.x 20305796 PMC2841346

[R59] Waldron M, Heath AC, Lynskey MT, Bucholz KK, Madden PA, & Martin NG (2011). Alcoholic marriage: Later start, sooner end. Alcoholism: Clinical and Experimental Research, 35, 632–642. 10.1111/j.1530-0277.2010.01381.x 21244438 PMC3066284

[R60] White W. (2006). Recovery across the life cycle. Alcoholism Treatment Quarterly, 24, 185–201. 10.1300/J020v24n01_11 PMC1526775 16892161

[R61] Witkiewitz K, Montes KS, Schwebel FJ, & Tucker JA (2020). What is recovery? Alcohol Research: Current Reviews, 40(3). 10.35946/arcr.v40.3.01 PMC7505137 32983748

[R62] Yamaguchi K, & Kandel DB (1985). On the resolution of role incompatibility: A life event history analysis of family roles and marijuana use. American Journal of Sociology, 90, 1284–1325. 10.1086/228211

