# Supplementary material for: A life span developmental investigation of marriage and problem-drinking reduction
Source: Dev Psychopathol. Author manuscript; Available in PMC 2024 Aug 1. (PMC10281208; doi:10.1017/S0954579422000827)
Supplement: 1 [file NIHMS1823241-supplement-1.docx]

**Online Supplement S1:**

**Detailed information regarding age and the age-based restructuring of waves into age bins**

Table S1.1

*Detailed information regarding AFDP and AHB ages, both in an original wave-based data structure and in the current study’s age-binned structure.*

| **AFDP (current sample N=577)** | | | | | | | | | | | | | | | | | | | | | | | | | | | | | |
| --- | --- | --- | --- | --- | --- | --- | --- | --- | --- | --- | --- | --- | --- | --- | --- | --- | --- | --- | --- | --- | --- | --- | --- | --- | --- | --- | --- | --- | --- |
| Wave 4  (*N*=527) | | | Wave 5  (*N*=516) | | | Wave 6  (*N*=507) | | | -- | | | Age bin 17-20 (*N*=324) | | | Age bin 21-23 (*N*=250) | | | Age bin 24-26 (*N*=330) | | | Age bin 27-30 (*N*=300) | | | Age bin 31-34 (*N*=281) | | | Age bin 35-39  (*N*=65) | | |
| Ages | N | % of N | Ages | n | % of N | Ages | N | % of N | -- | -- | -- | Ages | n | % of N | Ages | n | % of N | Ages | N | % of N | Ages | n | % of N | Ages | n | % of N | Ages | N | % of N |
| 17 | 8 | 1.5% | 21 | 1 | 0.2% | 27 | 2 | 0.4% | -- | -- | -- | 17 | 8 | 2.5% | 21 | 95 | 38.0% | 24 | 134 | 40.6% | 27 | 50 | 16.7% | 31 | 95 | 33.8% | 35 | 22 | 33.8% |
| 18 | 132 | 25.0% | 22 | 29 | 5.6% | 28 | 35 | 6.9% | -- | -- | -- | 18 | 132 | 40.7% | 22 | 72 | 28.8% | 25 | 107 | 32.4% | 28 | 70 | 23.3% | 32 | 83 | 29.5% | 36 | 20 | 30.8% |
| 19 | 91 | 17.3% | 23 | 60 | 11.6% | 29 | 58 | 11.4% | -- | -- | -- | 19 | 91 | 28.1% | 23 | 83 | 33.2% | 26 | 89 | 27.0% | 29 | 79 | 26.3% | 33 | 59 | 21.0% | 37 | 10 | 15.4% |
| 20 | 93 | 17.6% | 24 | 114 | 22.1% | 30 | 84 | 16.6% | -- | -- | -- | 20 | 93 | 28.7% |  |  |  |  |  |  | 30 | 101 | 33.7% | 34 | 44 | 15.7% | 38 | 8 | 12.3% |
| 21 | 94 | 17.8% | 25 | 91 | 17.6% | 31 | 82 | 16.2% | -- | -- | -- |  |  |  |  |  |  |  |  |  |  |  |  |  |  |  | 39 | 5 | 7.7% |
| 22 | 43 | 8.2% | 26 | 82 | 15.9% | 32 | 80 | 15.8% | -- | -- | -- |  |  |  |  |  |  |  |  |  |  |  |  |  |  |  |  |  |  |
| 23 | 23 | 4.4% | 27 | 48 | 9.3% | 33 | 57 | 11.2% | -- | -- | -- |  |  |  |  |  |  |  |  |  |  |  |  |  |  |  |  |  |  |
| 24 | 20 | 3.8% | 28 | 35 | 6.8% | 34 | 44 | 8.7% | -- | -- | -- |  |  |  |  |  |  |  |  |  |  |  |  |  |  |  |  |  |  |
| 25 | 16 | 3.0% | 29 | 21 | 4.1% | 35 | 22 | 4.3% | -- | -- | -- |  |  |  |  |  |  |  |  |  |  |  |  |  |  |  |  |  |  |
| 26 | 7 | 1.3% | 30 | 17 | 3.3% | 36 | 20 | 3.9% | -- | -- | -- |  |  |  |  |  |  |  |  |  |  |  |  |  |  |  |  |  |  |
|  |  |  | 31 | 13 | 2.5% | 37 | 10 | 2.0% | -- | -- | -- |  |  |  |  |  |  |  |  |  |  |  |  |  |  |  |  |  |  |
|  |  |  | 32 | 3 | 0.6% | 38 | 8 | 1.6% | -- | -- | -- |  |  |  |  |  |  |  |  |  |  |  |  |  |  |  |  |  |  |
|  |  |  | 33 | 2 | 0.4% | 39 | 5 | 1.0% | -- | -- | -- |  |  |  |  |  |  |  |  |  |  |  |  |  |  |  |  |  |  |
| **AHB (current sample N=441)** | | | | | | | | | | | | | | | | | | | | | | | | | | | | | |
| Wave 4  (*N*=432) | | | Wave 5  (*N*=423) | | | Wave 6  (*N*=380) | | | Wave 7  (*N*=355) | | | -- | | | Age bin 21-23 (*N*=426) | | | Age bin 24-26 (*N*=419) | | | Age bin 27-30 (*N*=378) | | | Age bin 31-34 (*N*=244) | | | Age bin 35-39 (*N*=123) | | |
| Ages | N | % of N | Ages | n | % of N | Ages | n | % of N | Ages | n | % of N | -- | -- | -- | Ages | n | % of N | Ages | N | % of N | Ages | n | % of N | Ages | n | % of N | Ages | n | % of N |
| 20 | 6 | 1.4% | 23 | 1 | 0.2% | 28 | 100 | 26.3% | 33 | 27 | 7.6% | -- | -- | -- | 21 | 327 | 76.8% | 24 | 264 | 63.0% | 27 | 4 | 1.1% | 31 | 5 | 2.0% | 35 | 109 | 88.6% |
| 21 | 327 | 74.7% | 24 | 261 | 61.7% | 29 | 215 | 56.6% | 34 | 207 | 58.3% | -- | -- | -- | 22 | 95 | 22.3% | 25 | 148 | 35.3% | 28 | 100 | 26.5% | 32 | 1 | 0.4% | 36 | 8 | 6.5% |
| 22 | 95 | 21.7% | 25 | 146 | 34.5% | 30 | 56 | 14.7% | 35 | 109 | 30.7% | -- | -- | -- | 23 | 4 | 0.9% | 26 | 7 | 1.7% | 29 | 217 | 57.4% | 33 | 30 | 12.3% | 37 | 6 | 4.9% |
| 23 | 3 | 0.7% | 26 | 7 | 1.7% | 31 | 5 | 1.3% | 36 | 7 | 2.0% | -- | -- | -- |  |  |  |  |  |  | 30 | 57 | 15.1% | 34 | 208 | 85.2% |  |  |  |
| 24 | 3 | 0.7% | 27 | 4 | 0.9% | 32 | 1 | 0.3% | 37 | 5 | 1.4% | -- | -- | -- |  |  |  |  |  |  |  |  |  |  |  |  |  |  |  |
| 25 | 2 | 0.5% | 29 | 2 | 0.5% | 33 | 2 | 0.5% |  |  |  | -- | -- | -- |  |  |  |  |  |  |  |  |  |  |  |  |  |  |  |
| 30 | 1 | 0.2% | 33 | 1 | 0.2% | 36 | 1 | 0.3% |  |  |  | -- | -- | -- |  |  |  |  |  |  |  |  |  |  |  |  |  |  |  |
| 34 | 1 | 0.2% | 37 | 1 | 0.2% |  |  |  |  |  |  | -- | -- | -- |  |  |  |  |  |  |  |  |  |  |  |  |  |  |  |

Figure S1.1

*Depiction of restructuring AFDP and AHB wave-based data into age-binned data*

| *AFDP* wave-based data | *AFDP* data restructured into age bins | |
| --- | --- | --- |
| 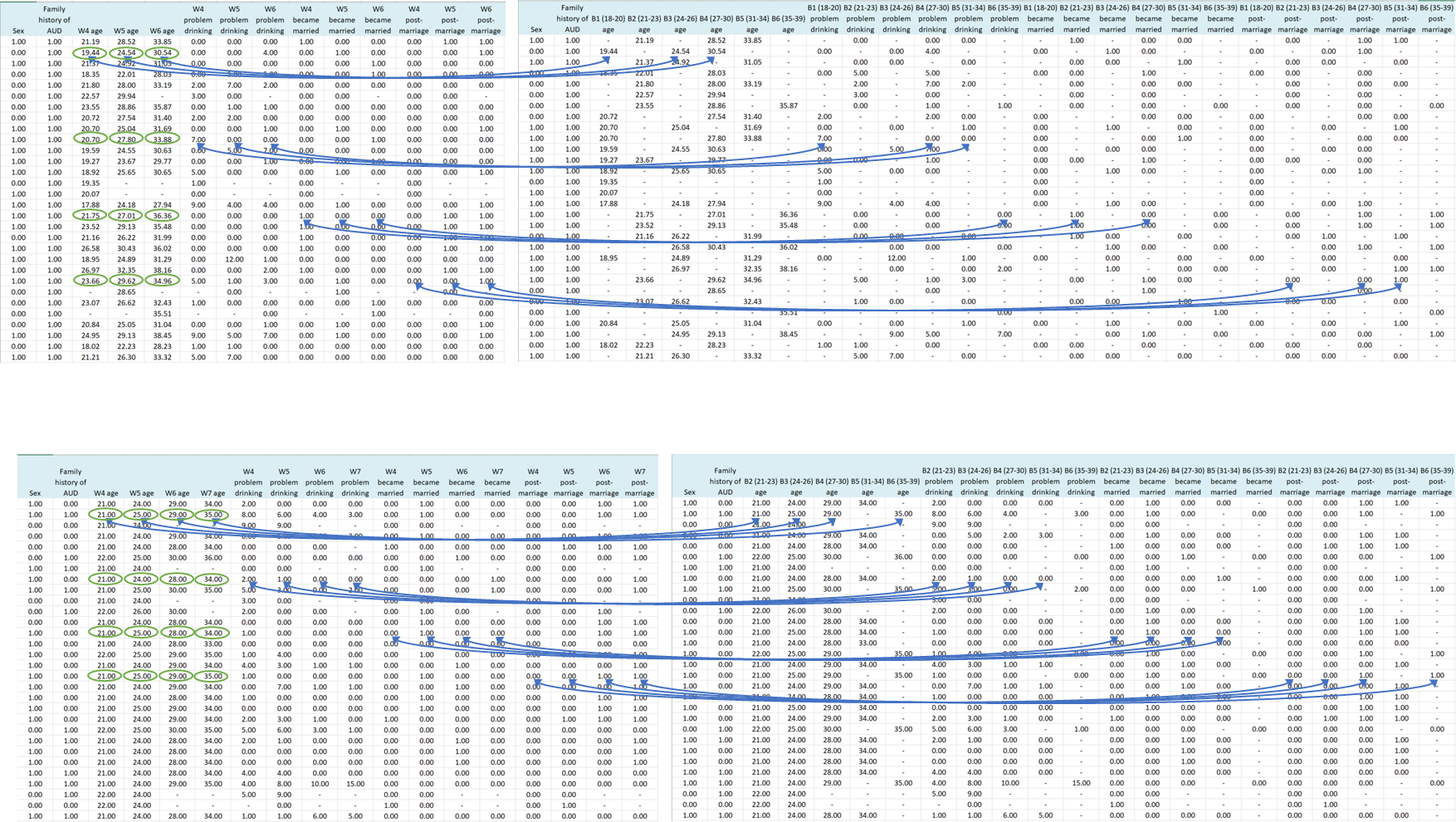 | 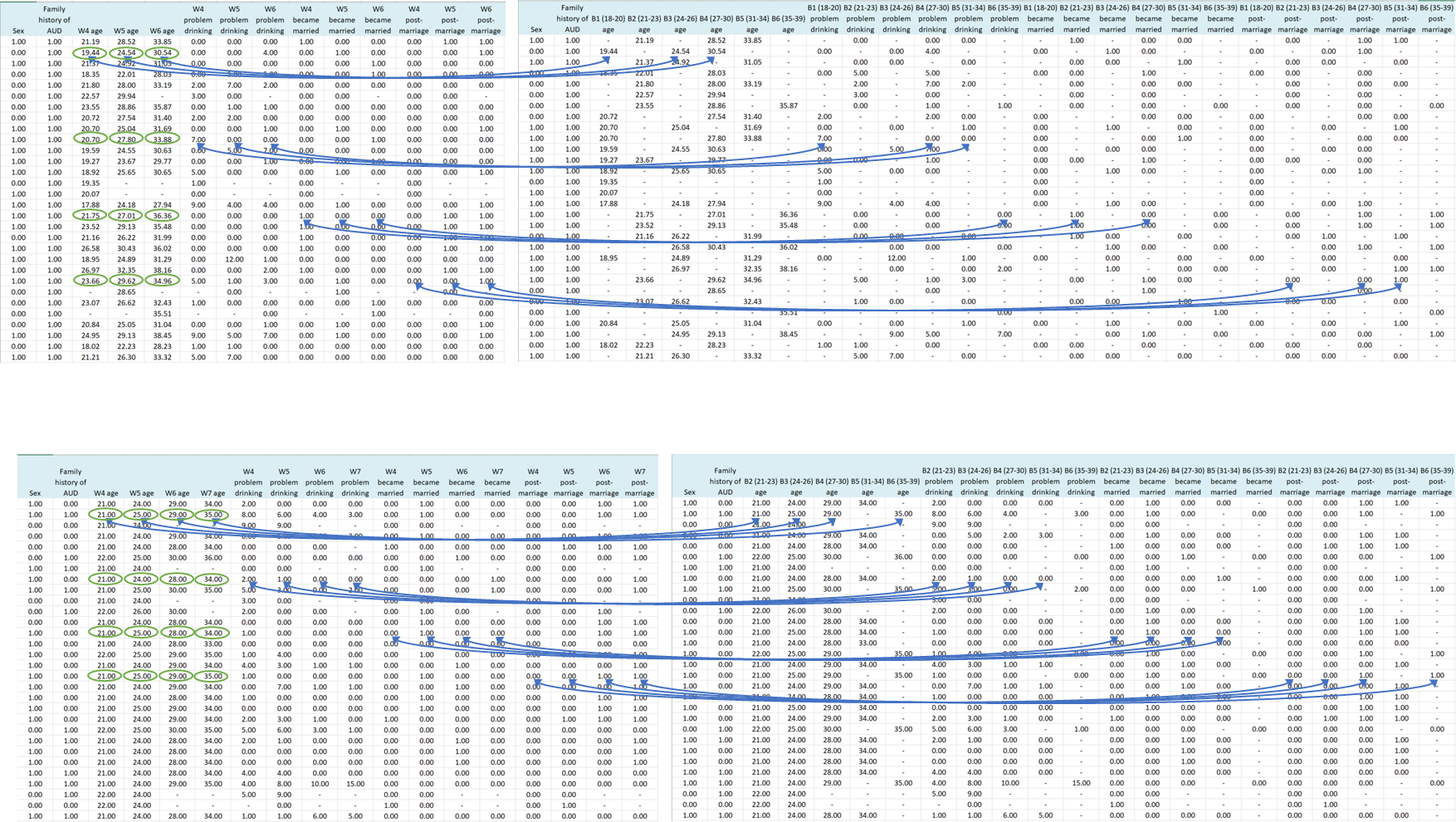 | |
| *AHB* wave-based data | | *AHB* data restructured into age bins |
| 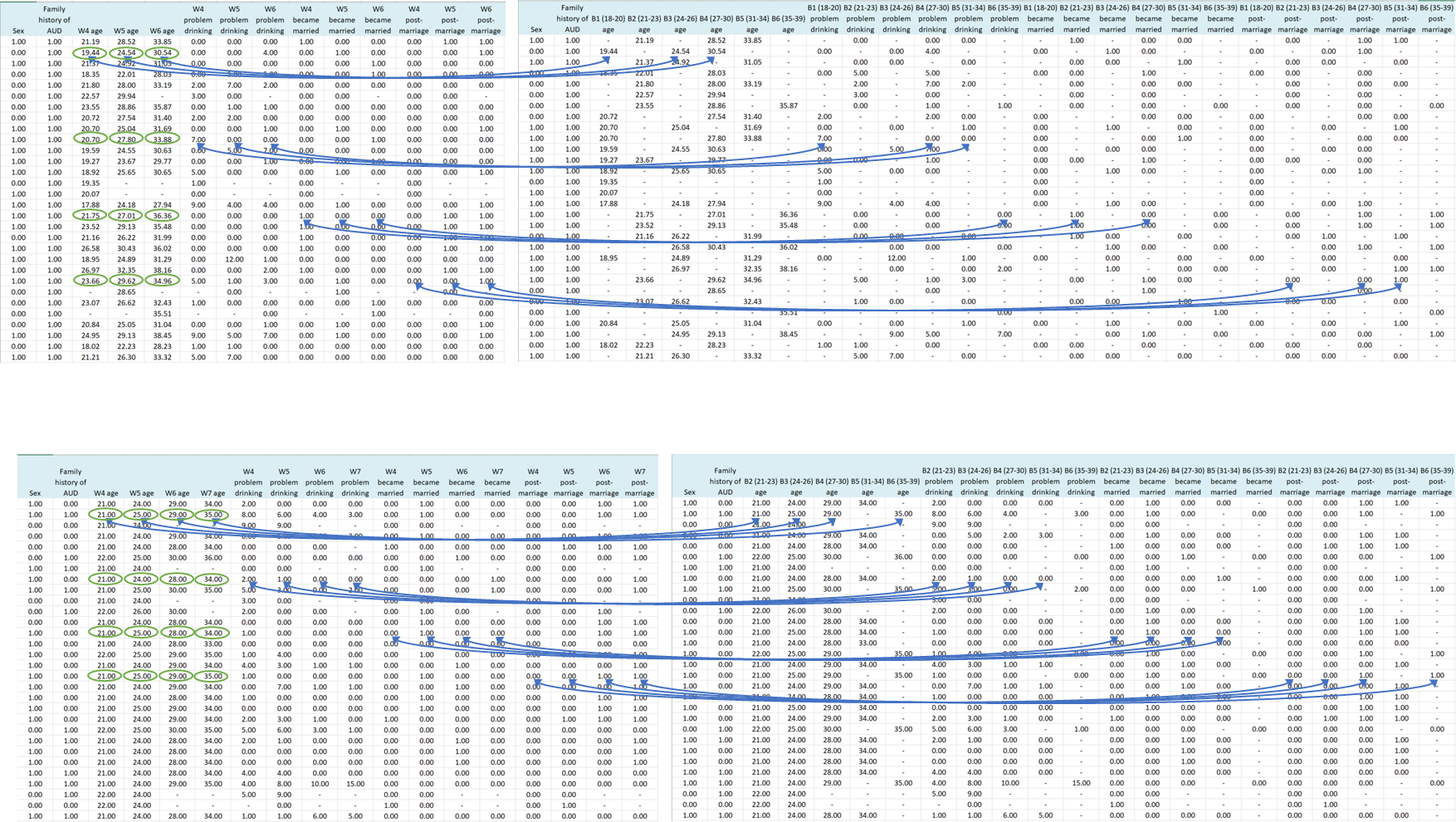 | | 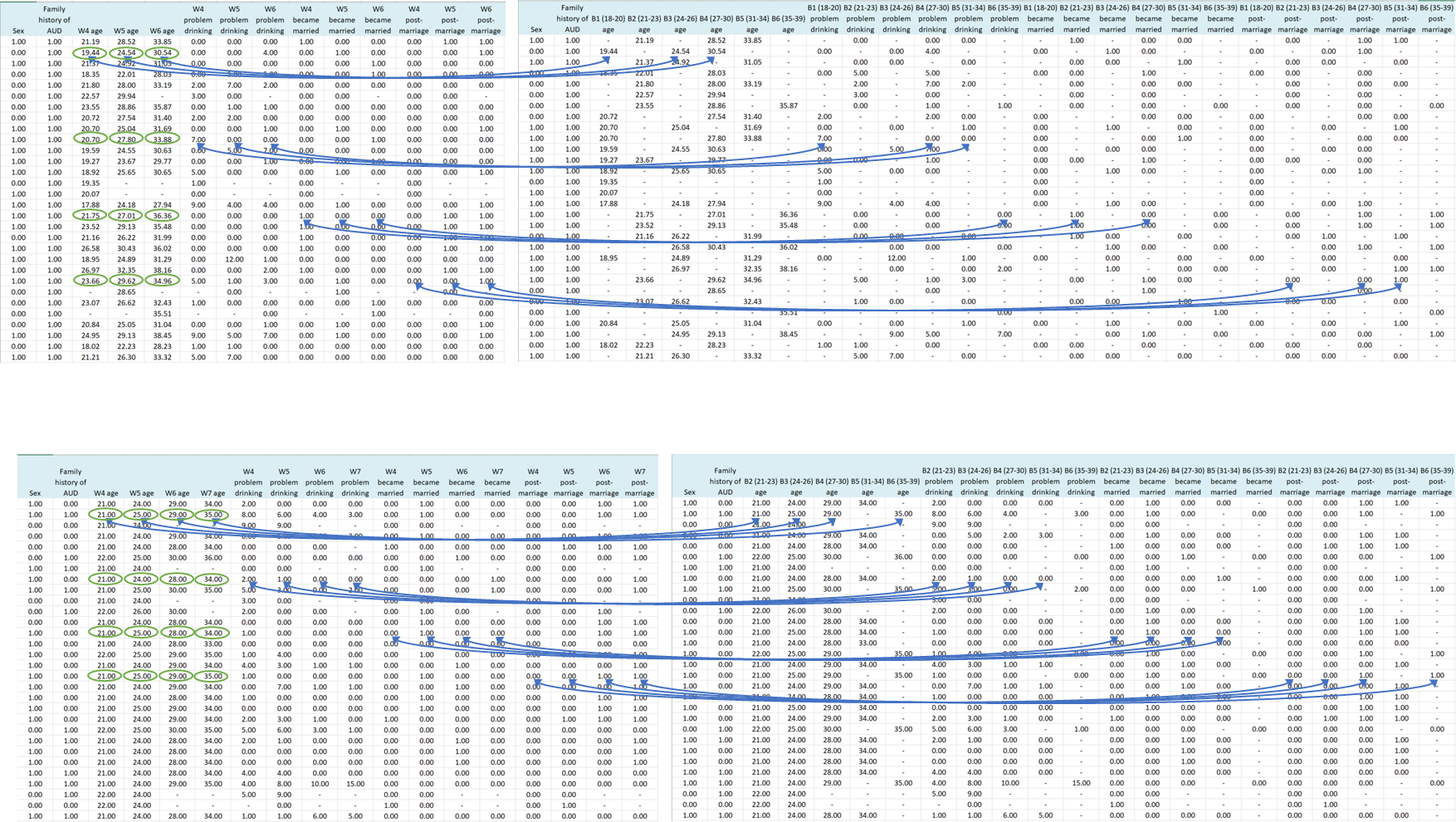 |

**Online Supplement S2:**

**Detailed information regarding the problem-drinking outcome variable**

Table S2.1

*Lists of AFDP and AHB dependence and consequence items used to index problem drinking, organized to illustrate content overlap between the two studies*

| AFDP problem-drinking items | AHB problem-drinking items |
| --- | --- |
| Using much larger amounts of alcohol than you expected to or over more days than you intended to |  |
| Tried to cut down on alcohol but found that you couldn't | After you have had several drinks, are you unable to stop drinking if you want |
|  | Try to cut back on your drinking |
| Needed larger amounts of alcohol to get an effect or could no longer get high on the amount | Needed larger amounts of alcohol to feel any effect or could no longer get high or drunk on the amount |
| Needed a drink just after you'd gotten up - that is, before breakfast | Felt like you needed a drink just after you'd gotten up (that is, before breakfast) |
| Had withdrawal symptoms because you stopped using alcohol or cut down on your alcohol use |  |
|  | Had "the shakes" after stopping or cutting down on drinking |
| Spent so much time arranging to get alcohol or having it on your mind that you had little time for anything else |  |
| Alcohol use caused you to neglect some of your usual responsibilities |  |
| Miss school or work because of your alcohol use | Not gone to work or missed classes at school because of drinking, a hangover, or an illness caused by drinking |
|  | Shown up late for work or school because of drinking, a hangover, or an illness caused by drinking |
|  | Neglect obligations, your family, your work or schoolwork for two or more days in a row because of drinking |
| Used alcohol enough so that you felt like you needed it or depended on it | Felt that you needed alcohol or were dependent on alcohol |
| Awakened the morning after using alcohol and could not remember a part of the evening before | Awakened the morning after a good bit of drinking and could not remember a part of the evening before |
| Ever gone on binges or benders where you kept drinking for a couple of days or more without sobering up | Go on a drinking binge or bender (that is, drinking for two or more days without sobering up) |
|  | Felt very sick to your stomach or thrown up after drinking |
| Felt guilty about your drinking | Felt guilty about your drinking |
| Get in trouble at school or work because of your alcohol use | Gotten into trouble at work or school because of drinking |
|  | Fired, suspended, expelled due to drinking |
| Have problems with your schoolwork or studying because of your alcohol use |  |
|  | Received a lower grade on an exam or paper than you should have because of your drinking |
| Alcohol use caused you to have financial problems |  |
| Get complaints from your family about your alcohol use | Spouse, boyfriend, girlfriend, parent(s), or other near relative complained to you about your drinking |
| Get complaints from your friends about your alcohol use |  |
|  | Lost friends (including boyfriends or girlfriends) because of your drinking |
|  | Drinking created problems between you and your spouse, boyfriend, girlfriend, or another near relative |
| Destroy property because of your alcohol use | Damaged property, set off a false alarm, or other things like that after you had been drinking |
| Get into a physical fight or do mean things because of your alcohol use | Gotten into physical fights when drinking |
| Alcohol use caused you to injure someone else |  |
| Alcohol use gotten you into sexual situations that you later regretted | Has drinking ever gotten you into sexual situations which you later regretted |
| Have an accident or injury because of your alcohol use |  |
| Drive a car or other motor vehicle when you knew you had too much alcohol to drive safely | Driven a car when you knew you had too much to drink to drive safely |
| Get arrested because of your alcohol use | Arrested for drunken driving, driving while intoxicated, or driving under the influence of alcohol |
|  | Arrested, even for a few hours, because of other drunken behavior |
| Pass out or faint because of your alcohol use |  |
|  | Had a headache (hangover) the morning after you had been drinking |
|  | Doctor ever told you that your drinking was harming your health |

Table S2.2

*Detailed descriptive information on the problem-drinking outcome variable, both in an original wave-based data structure and in the current study’s age-binned structure.*

| **AFDP (current sample N=577)** | | | | | | | | | | | | | | | | | | | | | | | | | | | | | |
| --- | --- | --- | --- | --- | --- | --- | --- | --- | --- | --- | --- | --- | --- | --- | --- | --- | --- | --- | --- | --- | --- | --- | --- | --- | --- | --- | --- | --- | --- |
| Wave 4  (*N*=527) | | | Wave 5  (*N*=516) | | | Wave 6  (*N*=507) | | | -- | | | Age bin 17-20 (*N*=324) | | | Age bin 21-23 (*N*=250) | | | Age bin 24-26 (*N*=330) | | | Age bin 27-30 (*N*=300) | | | Age bin 31-34 (*N*=281) | | | Age bin 35-39  (*N*=65) | | |
| mean=1.9336 | | | mean=1.3992 | | | mean=1.2623 | | |  | | | mean=1.9414 | | | mean=2.0600 | | | mean=1.3970 | | | mean=1.3100 | | | mean=1.1708 | | | mean=0.8308 | | |
| *SD*=3.16248 | | | *SD*=2.63868 | | | *SD*=2.74478 | | |  | | | *SD*=3.20984 | | | *SD*=3.14451 | | | *SD*=2.77253 | | | *SD*=2.46865 | | | *SD*=2.68182 | | | *SD*=2.56521 | | |
| skewness=2.342 | | | skewness=2.848 | | | skewness=3.455 | | |  | | | skewness=2.204 | | | skewness=2.178 | | | skewness=3.228 | | | skewness=3.458 | | | skewness=3.385 | | | skewness=4.377 | | |
| kurtosis=6.326 | | | kurtosis=9.844 | | | kurtosis=14.901 | | |  | | | kurtosis=5.025 | | | kurtosis=5.334 | | | kurtosis=13.235 | | | kurtosis=19.053 | | | kurtosis=12.897 | | | kurtosis=21.394 | | |
| Count | n | (%) | Count | n | (%) | Count | n | (%) | -- | -- | -- | Count | N | (%) | Count | n | (%) | Count | n | (%) | Count | n | (%) | Count | n | (%) | Count | n | (%) |
| 0 | 262 | (49.7%) | 0 | 305 | (59.1%) | 0 | 332 | (65.5%) |  |  |  | 0 | 167 | (51.5%) | 0 | 112 | (44.8%) | 0 | 196 | (59.4%) | 0 | 182 | (60.7%) | 0 | 191 | (68.0%) | 0 | 51 | (78.5%) |
| 1 | 85 | (16.1%) | 1 | 74 | (14.3%) | 1 | 62 | (12.2%) |  |  |  | 1 | 49 | (15.1%) | 1 | 47 | (18.8%) | 1 | 49 | (14.8%) | 1 | 35 | (11.7%) | 1 | 34 | (12.1%) | 1 | 7 | (10.8%) |
| 2 | 51 | (9.7%) | 2 | 44 | (8.5%) | 2 | 28 | (5.5%) |  |  |  | 2 | 28 | (8.6%) | 2 | 24 | (9.6%) | 2 | 29 | (8.8%) | 2 | 27 | (9.0%) | 2 | 14 | (5.0%) | 2 | 1 | (1.5%) |
| 3 | 29 | (5.5%) | 3 | 19 | (3.7%) | 3 | 20 | (3.9%) |  |  |  | 3 | 17 | (5.2%) | 3 | 16 | (6.4%) | 3 | 12 | (3.6%) | 3 | 13 | (4.3%) | 3 | 9 | (3.2%) | 3 | 1 | (1.5%) |
| 4 | 22 | (4.2%) | 4 | 21 | (4.1%) | 4 | 19 | (3.7%) |  |  |  | 4 | 15 | (4.6%) | 4 | 10 | (4.0%) | 4 | 11 | (3.3%) | 4 | 15 | (5.0%) | 4 | 10 | (3.6%) | 4 | 1 | (1.5%) |
| 5 | 10 | (1.9%) | 5 | 14 | (2.7%) | 5 | 15 | (3.0%) |  |  |  | 5 | 5 | (1.5%) | 5 | 7 | (2.8%) | 5 | 9 | (2.7%) | 5 | 10 | (3.3%) | 5 | 7 | (2.5%) | 5 | 1 | (1.5%) |
| 6 | 16 | (3.0%) | 6 | 9 | (1.7%) | 6 | 5 | (1.0%) |  |  |  | 6 | 8 | (2.5%) | 6 | 11 | (4.4%) | 6 | 4 | (1.2%) | 6 | 4 | (1.3%) | 6 | 3 | (1.1%) | 7 | 1 | (1.5%) |
| 7 | 14 | (2.7%) | 7 | 7 | (1.4%) | 7 | 5 | (1.0%) |  |  |  | 7 | 11 | (3.4%) | 7 | 4 | (1.6%) | 7 | 4 | (1.2%) | 7 | 5 | (1.7%) | 7 | 1 | (0.4%) | 10 | 1 | (1.5%) |
| 8 | 10 | (1.9%) | 8 | 7 | (1.4%) | 8 | 4 | (0.8%) |  |  |  | 8 | 5 | (1.5%) | 8 | 5 | (2.0%) | 8 | 5 | (1.5%) | 8 | 4 | (1.3%) | 8 | 2 | (0.7%) | 16 | 1 | (1.5%) |
| 9 | 5 | (0.9%) | 9 | 4 | (0.8%) | 9 | 3 | (0.6%) |  |  |  | 9 | 3 | (0.9%) | 9 | 3 | (1.2%) | 9 | 3 | (0.9%) | 9 | 2 | (0.7%) | 9 | 1 | (0.4%) |  |  |  |
| 10 | 5 | (0.9%) | 10 | 1 | (0.2%) | 10 | 4 | (0.8%) |  |  |  | 10 | 4 | (1.2%) | 10 | 2 | (0.8%) | 11 | 2 | (0.6%) | 10 | 1 | (0.3%) | 10 | 2 | (0.7%) |  |  |  |
| 11 | 5 | (0.9%) | 11 | 3 | (0.6%) | 11 | 3 | (0.6%) |  |  |  | 11 | 3 | (0.9%) | 11 | 3 | (1.2%) | 12 | 3 | (0.9%) | 14 | 1 | (0.3%) | 11 | 3 | (1.1%) |  |  |  |
| 12 | 3 | (0.6%) | 12 | 4 | (0.8%) | 14 | 2 | (0.4%) |  |  |  | 12 | 2 | (0.6%) | 12 | 2 | (0.8%) | 15 | 1 | (0.3%) | 22 | 1 | (0.3%) | 14 | 1 | (0.4%) |  |  |  |
| 13 | 5 | (0.9%) | 13 | 1 | (0.2%) | 16 | 4 | (0.8%) |  |  |  | 13 | 4 | (1.2%) | 13 | 2 | (0.8%) | 16 | 1 | (0.3%) |  |  |  | 16 | 3 | (1.1%) |  |  |  |
| 14 | 3 | (0.6%) | 15 | 1 | (0.2%) | 22 | 1 | (0.2%) |  |  |  | 14 | 2 | (0.6%) | 14 | 1 | (0.4%) | 21 | 1 | (0.3%) |  |  |  |  |  |  |  |  |  |
| 19 | 1 | (0.2%) | 16 | 1 | (0.2%) |  |  |  |  |  |  | 19 | 1 | (0.3%) | 19 | 1 | (0.4%) |  |  |  |  |  |  |  |  |  |  |  |  |
| 21 | 1 | (0.2%) | 19 | 1 | (0.2%) |  |  |  |  |  |  |  |  |  |  |  |  |  |  |  |  |  |  |  |  |  |  |  |  |
| **AHB (current sample N=441)** | | | | | | | | | | | | | | | | | | | | | | | | | | | | | |
| Wave 4  (*N*=432) | | | Wave 5  (*N*=423) | | | Wave 6  (*N*=380) | | | Wave 7  (*N*=355) | | | -- | | | Age bin 21-23 (*N*=426) | | | Age bin 24-26 (*N*=419) | | | Age bin 27-30 (*N*=378) | | | Age bin 31-34 (*N*=244) | | | Age bin 35-39 (*N*=123) | | |
| mean=2.7064 | | | mean=1.7043 | | | mean=1.0464 | | | mean=1.0087 | | |  | | | mean=2.7217 | | | mean=1.6877 | | | mean=1.0220 | | | mean=1.0210 | | | mean=1.1949 | | |
| *SD*=3.07770 | | | *SD*=2.50524 | | | *SD*=1.95722 | | | *SD*=1.93272 | | |  | | | *SD*=3.09471 | | | *SD*=2.49110 | | | *SD*=1.91545 | | | *SD*=1.95832 | | | *SD*=2.14937 | | |
| skewness=2.044 | | | skewness=2.601 | | | skewness=2.893 | | | skewness=2.855 | | |  | | | skewness=2.046 | | | skewness=2.645 | | | skewness=2.911 | | | skewness=2.336 | | | skewness=3.305 | | |
| kurtosis=6.169 | | | kurtosis=10.930 | | | kurtosis=11.194 | | | kurtosis=11.049 | | |  | | | kurtosis=6.163 | | | kurtosis=11.323 | | | kurtosis=11.572 | | | kurtosis=5.163 | | | kurtosis=15.779 | | |
| Count | n | (%) | Count | n | (%) | Count | n | (%) | Count | n | (%) | -- | -- | -- | Count | n | (%) | Count | n | (%) | Count | n | (%) | Count | n | (%) | Count | n | (%) |
| 0 | 110 | (26.4%) | 0 | 190 | (43.9%) | 0 | 241 | (62%) | 0 | 233 | (64.6%) |  |  |  | 0 | 113 | (26.2%) | 0 | 188 | (44.1%) | 0 | 241 | (62.5%) | 0 | 183 | (65.8%) | 0 | 74 | (58.6%) |
| 1 | 85 | (19.5%) | 1 | 89 | (21.4%) | 1 | 56 | (15.3%) | 1 | 44 | (12.8%) |  |  |  | 1 | 82 | (19.3%) | 1 | 89 | (21.5%) | 1 | 56 | (15.4%) | 1 | 28 | (11.8%) | 1 | 17 | (14.4%) |
| 2 | 54 | (12.4%) | 2 | 45 | (10.8%) | 2 | 25 | (6.8%) | 2 | 27 | (7.8%) |  |  |  | 2 | 51 | (12.0%) | 2 | 45 | (10.9%) | 2 | 24 | (6.6%) | 2 | 20 | (8.4%) | 2 | 9 | (7.6%) |
| 3 | 56 | (12.8%) | 3 | 22 | (5.3%) | 3 | 24 | (6.6%) | 3 | 16 | (4.6%) |  |  |  | 3 | 55 | (13.0%) | 3 | 22 | (5.3%) | 3 | 25 | (6.9%) | 3 | 8 | (3.4%) | 3 | 8 | (6.8%) |
| 4 | 39 | (8.9%) | 4 | 24 | (5.8%) | 4 | 8 | (2.2%) | 4 | 12 | (3.5%) |  |  |  | 4 | 39 | (9.2%) | 4 | 23 | (5.6%) | 4 | 7 | (1.9%) | 4 | 4 | (1.7%) | 4 | 10 | (8.5%) |
| 5 | 27 | (6.2%) | 5 | 17 | (4.1%) | 5 | 12 | (3.3%) | 5 | 9 | (2.6%) |  |  |  | 5 | 27 | (6.4%) | 5 | 17 | (4.1%) | 5 | 11 | (3.0%) | 5 | 8 | (3.4%) | 5 | 2 | (1.7%) |
| 6 | 16 | (3.7%) | 6 | 11 | (2.6%) | 6 | 6 | (1.6%) | 6 | 4 | (1.2%) |  |  |  | 6 | 16 | (3.8%) | 6 | 11 | (2.7%) | 6 | 6 | (1.6%) | 6 | 4 | (1.7%) | 7 | 1 | (0.8%) |
| 7 | 17 | (3.9%) | 7 | 14 | (3.4%) | 7 | 1 | (0.3%) | 7 | 5 | (1.4%) |  |  |  | 7 | 15 | (3.5%) | 7 | 14 | (3.4%) | 7 | 2 | (0.5%) | 7 | 4 | (1.7%) | 10 | 1 | (0.8%) |
| 8 | 5 | (1.1%) | 8 | 2 | (0.5%) | 8 | 2 | (0.5%) | 8 | 2 | (0.6%) |  |  |  | 8 | 5 | (1.2%) | 8 | 1 | (0.2%) | 8 | 2 | (0.5%) | 8 | 3 | (1.3%) | 15 | 1 | (0.8%) |
| 9 | 5 | (1.1%) | 9 | 5 | (1.2%) | 9 | 1 | (0.3%) | 9 | 1 | (0.3%) |  |  |  | 9 | 5 | (1.2%) | 9 | 5 | (1.2%) | 9 | 1 | (0.3%) | 9 | 1 | (0.4%) |  |  |  |
| 10 | 5 | (1.1%) | 10 | 2 | (0.5%) | 10 | 3 | (0.8%) | 10 | 1 | (0.3%) |  |  |  | 10 | 5 | (1.2%) | 10 | 2 | (0.5%) | 10 | 2 | (0.5%) | 10 | 1 | (0.4%) |  |  |  |
| 11 | 5 | (1.1%) | 19 | 2 | (0.5%) | 15 | 1 | (0.3%) | 15 | 1 | (0.3%) |  |  |  | 11 | 5 | (1.2%) | 19 | 2 | (0.5%) | 15 | 1 | (0.3%) |  |  |  |  |  |  |
| 12 | 3 | (0.7%) |  |  |  |  |  |  |  |  |  |  |  |  | 12 | 3 | (0.7%) |  |  |  |  |  |  |  |  |  |  |  |  |
| 14 | 1 | (0.2%) |  |  |  |  |  |  |  |  |  |  |  |  | 14 | 1 | (0.2%) |  |  |  |  |  |  |  |  |  |  |  |  |
| 17 | 2 | (0.5%) |  |  |  |  |  |  |  |  |  |  |  |  | 17 | 2 | (0.5%) |  |  |  |  |  |  |  |  |  |  |  |  |
| 18 | 1 | (0.2%) |  |  |  |  |  |  |  |  |  |  |  |  | 18 | 1 | (0.2%) |  |  |  |  |  |  |  |  |  |  |  |  |
| 21 | 1 | (0.2%) |  |  |  |  |  |  |  |  |  |  |  |  | 21 | 1 | (0.2%) |  |  |  |  |  |  |  |  |  |  |  |  |

**Online Supplement S3:**

**Showing that estimating free-curve negative binomial models for both AFDP and AHB yielded similar results to those in the main manuscript where different best-fitting models were retained for AFDP (linear negative binomial) and AHB (free curve Poisson)**

Table S3.1: *Results of supplemental analyses with free-curve negative binomial modeling for both AFDP and AHB*

|  | AFDP free-curve negative binomial model | | AHB free-curve negative binomial model | |
| --- | --- | --- | --- | --- |
|  | Estimates | p-value | Estimates | p-value |
| Effects on age 17-20 Problem Drinking |  |  |  |  |
| Sex (0=male; 1=female) | -0.935 | 0.000 | -- | -- |
| Familial AUD (0=neg.; 1=pos.) | 0.471 | 0.030 | -- | -- |
| Effects on age 21-23 Problem Drinking |  |  |  |  |
| **Became married by age 21-23** | **-1.201** | **0.003** | **--** | **--** |
| Sex (0=male; 1=female) | -0.569 | 0.000 | -0.690 | 0.000 |
| Familial AUD (0=neg.; 1=pos.) | 0.442 | 0.000 | 0.379 | 0.001 |
| Effects on age 24-26 Problem Drinking |  |  |  |  |
| **Became married by age 24-26** | **-0.777** | **0.000** | **-0.455** | **0.001** |
| Sex (0=male; 1=female) | -0.231 | 0.157 | -0.601 | 0.000 |
| Familial AUD (0=neg.; 1=pos.) | 0.194 | 0.190 | 0.299 | 0.012 |
| Effects on age 27-30 Problem Drinking |  |  |  |  |
| **Became married by age 27-30** | **-0.516** | **0.025** | **-0.392** | **0.001** |
| Previously married by age 27-30 | -0.341 | 0.298 | -0.606 | 0.000 |
| Sex (0=male; 1=female) | -0.781 | 0.000 | -0.409 | 0.001 |
| Familial AUD (0=neg.; 1=pos.) | 0.283 | 0.116 | 0.257 | 0.036 |
| Effects on age 31-34 Problem Drinking |  |  |  |  |
| **Became married by age 31-34** | **-0.433** | **0.010** | **0.026** | **0.886** |
| Previously married by age 31-34 | -0.266 | 0.131 | -0.435 | 0.003 |
| Sex (0=male; 1=female) | -0.480 | 0.003 | -0.458 | 0.002 |
| Familial AUD (0=neg.; 1=pos.) | 0.278 | 0.059 | 0.397 | 0.005 |
| Effects on age 35-39 Problem Drinking |  |  |  |  |
| **Became married by age 35-39** | **-0.113** | **0.841** | **0.485** | **0.097** |
| Previously married by age 35-39 | -0.640 | 0.157 | -0.572 | 0.031 |
| Sex (0=male; 1=female) | -0.200 | 0.672 | -0.367 | 0.188 |
| Familial AUD (0=neg.; 1=pos.) | -0.035 | 0.930 | -0.059 | 0.820 |
| **Wald χ^2^ test of omnibus differences among age bins in “became married” effects** | **χ^2^(4)=6.855** | **0.144** | **χ^2^(3)=9.923** | **0.019** |
| **Wald χ^2^ test of linear age-related change in “became married” effects across age bins^1^** | **χ^2^(1)=4.412** | **0.036** | **χ^2^(1)=4.510** | **0.034** |
| Intercept factor loadings |  |  |  |  |
| Age 17-20 Problem Drinking | @1 | -- | -- | -- |
| Age 21-23 Problem Drinking | @1 | -- | @1 | -- |
| Age 24-26 Problem Drinking | @1 | -- | @1 | -- |
| Age 27-30 Problem Drinking | @1 | -- | @1 | -- |
| Age 31-34 Problem Drinking | @1 | -- | @1 | -- |
| Age 35-39 Problem Drinking | @1 | -- | @1 | -- |
| Slope factor loadings |  |  |  |  |
| Age 17-20 Problem Drinking | @0 | -- | -- | -- |
| Age 21-23 Problem Drinking | @1 | -- | @0 | -- |
| Age 24-26 Problem Drinking | 1.338 | 0.000 | @1 | -- |
| Age 27-30 Problem Drinking | 0.351 | 0.301 | 2.309 | 0.000 |
| Age 31-34 Problem Drinking | 1.510 | 0.000 | 2.778 | 0.002 |
| Age 35-39 Problem Drinking | 1.602 | 0.068 | 1.200 | 0.088 |
| Growth factor means |  |  |  |  |
| Intercept factor | 0.477 | 0.013 | 0.752 | 0.000 |
| Slope factor | -0.616 | 0.001 | -0.477 | 0.000 |
| Growth factor variances |  |  |  |  |
| Intercept factor | 0.579 | 0.006 | 0.739 | 0.000 |
| Slope factor | 0.294 | 0.075 | 0.172 | 0.058 |
| Intercept factor with slope factor | 0.403 | 0.000 | 0.103 | 0.067 |
| Negative binomial dispersion parameters for growth-factor indicators |  |  |  |  |
| Age 17-20 Problem Drinking | 1.700 | 0.000 | -- | -- |
| Age 21-23 Problem Drinking | 0.000 | 1.000 | 0.013 | 0.902 |
| Age 24-26 Problem Drinking | 0.777 | 0.031 | 0.223 | 0.026 |
| Age 27-30 Problem Drinking | 1.748 | 0.000 | 0.014 | 0.949 |
| Age 31-34 Problem Drinking | 1.185 | 0.008 | 0.195 | 0.615 |
| Age 35-39 Problem Drinking | 3.373 | 0.054 | 0.457 | 0.066 |
| Intercepts of growth-factor indicators |  |  |  |  |
| Age 17-20 Problem Drinking | @0 | -- | -- | -- |
| Age 21-23 Problem Drinking | @0 | -- | @0 | -- |
| Age 24-26 Problem Drinking | @0 | -- | @0 | -- |
| Age 27-30 Problem Drinking | @0 | -- | @0 | -- |
| Age 31-34 Problem Drinking | @0 | -- | @0 | -- |
| Age 35-39 Problem Drinking | @0 | -- | @0 | -- |

*Note.* Reference group: never married. The “@” symbol indicates that a parameter was constrained to the given value rather than freely estimated.

**Online Supplement S4:**

Table S4.1

*Results of ADFP and AHB models testing age variability in effects of marriage on problem-drinking reductions separately among males and females*

|  | AFDP models  (linear NB) | | | | AHB models  (free-curve Poisson) | | | |
| --- | --- | --- | --- | --- | --- | --- | --- | --- |
|  | Males | | Females | | Males | | Females | |
|  | Estimates | p-value | Estimates | p-value | Estimates | p-value | Estimates | p-value |
| Effects on age 17-20 problem drinking |  |  |  |  |  |  |  |  |
| Familial AUD (0=negative; 1=positive) | 0.329 | 0.038 | 0.601 | 0.094 | -- | -- | -- | -- |
| Effects on age 21-23 problem drinking |  |  |  |  |  |  |  |  |
| **Became married by age 21-23** | **-2.045** | **0.000** | **-1.644** | **0.047** | **--** | **--** | **--** | **--** |
| Familial AUD (0=negative; 1=positive) | 0.459 | 0.000 | 0.572 | 0.076 | 0.375 | 0.011 | 0.360 | 0.026 |
| Effects on age 24-26 problem drinking |  |  |  |  |  |  |  |  |
| **Became married by age 24-26** | **-0.932** | **0.001** | **-1.373** | **0.001** | **-0.480** | **0.034** | **-0.548** | **0.019** |
| Familial AUD (0=negative; 1=positive) | 0.192 | 0.262 | 0.518 | 0.227 | 0.437 | 0.021 | 0.287 | 0.168 |
| Effects on age 27-30 problem drinking |  |  |  |  |  |  |  |  |
| **Became married by age 27-30** | **-0.247** | **0.352** | **-1.129** | **0.012** | **-0.403** | **0.035** | **-0.527** | **0.092** |
| Previously married by age 27-30 | 0.364 | 0.251 | -0.965 | 0.078 | -0.760 | 0.004 | -0.942 | 0.012 |
| Familial AUD (0=negative; 1=positive) | 0.485 | 0.010 | 0.581 | 0.091 | 0.170 | 0.495 | 0.809 | 0.010 |
| Effects on age 31-34 problem drinking |  |  |  |  |  |  |  |  |
| **Became married by age 31-34** | **-0.365** | **0.130** | **-1.779** | **0.004** | **-0.384** | **0.451** | **0.056** | **0.912** |
| Previously married by age 31-34 | -0.373 | 0.155 | -0.733 | 0.150 | -0.818 | 0.001 | -0.368 | 0.366 |
| Familial AUD (0=negative; 1=positive) | 0.385 | 0.061 | 0.523 | 0.297 | 0.464 | 0.124 | 0.699 | 0.081 |
| Effects on age 35-39 problem drinking |  |  |  |  |  |  |  |  |
| **Became married by age 35-39** | **0.125** | **0.782** | **--** | **--** | **0.568** | **0.088** | **0.926** | **0.272** |
| Previously married by age 35-39 | -0.440 | 0.210 | -- | -- | -0.927 | 0.001 | -0.260 | 0.640 |
| Familial AUD (0=negative; 1=positive) | -0.990 | 0.306 | -- | -- | -0.513 | 0.095 | 0.771 | 0.109 |
| **Wald χ^2^ *omnibus* test of age moderation of the “became married” effect** | **χ^2^(4)=37.00 (p<.001)** | | **χ^2^(3)=1.04 (p=.792)** | | **χ^2^(3)=7.55 (p=.056)** | | **χ^2^(3)=3.01 (p=.390)** | |
| **Wald χ^2^ *linear* test of age moderation of the “became married” effect** | **χ^2^(1)=11.67 (p<.001)** | | **χ^2^(1)=0.03 (p=.888)** | | **χ^2^(1)=3.91 (p=.048)** | | **χ^2^(1)=1.07 (p=.301)** | |
| Growth factor means |  |  |  |  |  |  |  |  |
| Intercept factor | 0.371 | 0.001 | -0.502 | 0.031 | -1.046 | 0.000 | -1.364 | 0.000 |
| Slope factor | -1.145 | 0.000 | -0.689 | 0.298 | 0.840 | 0.000 | 0.087 | 0.564 |
| Growth factor covariance |  |  |  |  |  |  |  |  |
| Intercept factor with slope factor | 0.120 | 0.421 | 0.989 | 0.000 | 0.119 | 0.421 | 0.346 | 0.049 |

**Online Supplement S5:**

**Ruling out the confounding of recency of marriage with younger age as an alternative explanation for our evidence of age as a moderator of marriage effects**

Table S5.1 below does show a pattern of greater recency of marriage among younger “became married” participants. Thus, if recency of marriage is also associated with stronger effects of marriage, it would represent a plausible alternative explanation to our finding of stronger marriage effects at younger ages. However, in the supplemental variation of our primary analysis in Table S4.2 that parsed “became married” groups into high-recency and low-recency subgroups, there was little evidence for stronger effects of more recent marriages. In fact, across the age bins, it more often appeared that *less* recency was associated with a slightly stronger “became married” effect (the opposite of what would be required for recency to represent a plausible alternative explanation). This rules out the confound of marital recency with younger age as an alternative explanation for our primary finding of greater marriage effects at younger ages.

Table S5.1

*Contrasting the “became married” groups across the age bins on recency of marriage (i.e., years since marriage)*

|  | **AFDP “Became Married” Groups at Each Age Bin** | | | | |
| --- | --- | --- | --- | --- | --- |
|  | Became married group at age bin 21-23 (*n*=250) | Became married group at age bin 24-26 (*n*=330) | Became married group at age bin 27-30 (*n*=300) | Became married group at age bin 31-34 (*n*=281) | Became married group at age bin 35-39  (*n*=65) |
| Years since marriage | mean=2.60 (*SD*=1.45) | mean=3.69 (*SD*=2.34) | mean=4.45 (*SD*=2.13) | mean=4.78 (*SD*=2.72) | Mean=5.15 (*SD*=3.16) |

*Note.* Data on age of marriage are available only in AFDP, so recency can only be assessed in AFDP.

*Note.* Years since marriage was computed as the participant’s age at the age bin when classified as “became married” minus the participant’s reported age of marriage.

Table S5.2

*Supplemental variation of the primary AFDP model testing two separate “became married” effects differentiated on marital recency at each age bin*

|  | Estimates | p-value |
| --- | --- | --- |
| Effects on age 17-20 problem drinking |  |  |
| Sex (0=male; 1=female) | -0.833 | 0.000 |
| Familial AUD (0=negative; 1=positive) | 0.423 | 0.029 |
| Effects on age 21-23 problem drinking |  |  |
| **High-recency became married by age 21-23** | **-1.315** | **0.018** |
| **Low-recency became married by age 21-23** | **-1.656** | **0.002** |
| Sex (0=male; 1=female) | -0.690 | 0.000 |
| Familial AUD (0=negative; 1=positive) | 0.462 | 0.001 |
| Effects on age 24-26 problem drinking |  |  |
| **High-recency became married by age 24-26** | **-0.733** | **0.002** |
| **Low-recency became married by age 24-26** | **-1.072** | **0.000** |
| Sex (0=male; 1=female) | -0.237 | 0.223 |
| Familial AUD (0=negative; 1=positive) | 0.185 | 0.267 |
| Effects on age 27-30 problem drinking |  |  |
| **High-recency became married by age 27-30** | **-0.573** | **0.101** |
| **Low-recency became married by age 27-30** | **-0.291** | **0.128** |
| Previously married by age 27-30 | -0.168 | 0.544 |
| Sex (0=male; 1=female) | -0.559 | 0.001 |
| Familial AUD (0=negative; 1=positive) | 0.378 | 0.010 |
| Effects on age 31-34 problem drinking |  |  |
| **High-recency became married by age 31-34** | **-0.469** | **0.068** |
| **Low-recency became married by age 31-34** | **-0.404** | **0.041** |
| Previously married by age 31-34 | -0.238 | 0.173 |
| Sex (0=male; 1=female) | -0.463 | 0.007 |
| Familial AUD (0=negative; 1=positive) | 0.287 | 0.111 |
| Effects on age 35-39 problem drinking |  |  |
| **High-recency became married by age 35-39** | **0.309** | **0.637** |
| **Low-recency became married by age 35-39** | **-0.563** | **0.290** |
| Previously married by age 35-39 | -0.506 | 0.175 |
| Sex (0=male; 1=female) | -0.030 | 0.941 |
| Familial AUD (0=negative; 1=positive) | -0.069 | 0.871 |
| Growth factor means |  |  |
| Intercept factor | 0.407 | 0.024 |
| Slope factor | -1.457 | 0.013 |
| Growth factor covariance |  |  |
| Intercept factor with slope factor | 0.593 | 0.367 |
| Intercept factor loadings |  |  |
| Age 17-20 problem drinking | @1 | -- |
| Age 21-23 problem drinking | @1 | -- |
| Age 24-26 problem drinking | @1 | -- |
| Age 27-30 problem drinking | @1 | -- |
| Age 31-34 problem drinking | @1 | -- |
| Age 35-39 problem drinking | @1 | -- |
| Slope factor loadings |  |  |
| Age 17-20 problem drinking | @0 | -- |
| Age 21-23 problem drinking | @0.17 | -- |
| Age 24-26 problem drinking | @0.33 | -- |
| Age 27-30 problem drinking | @0.50 | -- |
| Age 31-34 problem drinking | @0.69 | -- |
| Age 35-39 problem drinking | @1 | -- |

*Note.* “High-recency became married” participants at a given age bin were those who were in our original became married group at that age bin *and had become married less than three years earlier than their recorded age at that age bin*. “Low-recency became married” participants at a given age bin were those who were in our original became married group at that age bin *and had become married more than three years earlier than their recorded age at that age bin.* Unstandardized estimates are reported. The “@” symbol indicates that a parameter was constrained to the given value rather than freely estimated.

**Online Supplement S6:**

**Other lifecourse events in AFDP and AHB**

Table S6.1

*Prevalences of other lifecourse events in AFDP across waves and age bins*

|  | Wave 4 | | Wave 5 | | Wave 6 | | -- | | Age bin 17-20 | | Age bin 21-23 | | Age bin 24-26 | | Age bin 27-30 | | Age bin 31-34 | | Age bin 35-39 | |
| --- | --- | --- | --- | --- | --- | --- | --- | --- | --- | --- | --- | --- | --- | --- | --- | --- | --- | --- | --- | --- |
|  | N | % | n | % | n | % | n | % | n | % | n | % | N | % | n | % | n | % | n | % |
| Marital status |  |  |  |  |  |  |  |  |  |  |  |  |  |  |  |  |  |  |  |  |
| Never married or divorced | 590 | 80.5 | 389 | 51.8 | 266 | 36.1 | -- | -- | 361 | 93.3 | 245 | 71.2 | 266 | 56.5 | 182 | 42.8 | 144 | 33.7 | 47 | 28.5 |
| never married | -- | -- | -- | -- | -- | -- | -- | -- | -- | -- | -- | -- | -- | -- | -- | -- | -- | -- | -- | -- |
| divorced | -- | -- | -- | -- | -- | -- | -- | -- | -- | -- | -- | -- | -- | -- | -- | -- | -- | -- | -- | -- |
| Separated | 14 | 1.9 | 17 | 2.3 | 16 | 2.2 | -- | -- | 6 | 1.6 | 8 | 2.3 | 10 | 2.1 | 9 | 2.1 | 13 | 3.0 | 1 | 0.6 |
| Engaged | -- | -- | 35 | 4.7 | 32 | 4.3 | -- | -- | 0 | 0.0 | 6 | 1.7 | 19 | 4.0 | 23 | 5.4 | 13 | 3.0 | 6 | 3.6 |
| Married | 129 | 17.6 | 309 | 41.1 | 420 | 57 | -- | -- | 20 | 5.2 | 85 | 24.7 | 176 | 37.4 | 211 | 49.6 | 255 | 59.7 | 109 | 66.1 |
| Number of children |  |  |  |  |  |  |  |  |  |  |  |  |  |  |  |  |  |  |  |  |
| 0 | -- | -- | 120 | 15.9 | 19 | 3.8 | -- | -- | -- | -- | 14 | 13.1 | 63 | 17.3 | 40 | 11.9 | 20 | 6.2 | 2 | 1.5 |
| 1 | -- | -- | 114 | 15.1 | 119 | 23.5 | -- | -- | -- | -- | 4 | 3.7 | 35 | 9.6 | 92 | 27.4 | 84 | 26.0 | 18 | 13.8 |
| 2 | -- | -- | 47 | 6.2 | 192 | 37.9 | -- | -- | -- | -- | 2 | 1.9 | 11 | 3.0 | 66 | 19.6 | 110 | 34.1 | 50 | 38.5 |
| 3 | -- | -- | 13 | 1.7 | 105 | 20.8 | -- | -- | -- | -- | 0 | 0.0 | 1 | 0.3 | 26 | 7.7 | 61 | 18.9 | 30 | 23.1 |
| 4 | -- | -- | 1 | 0.1 | 49 | 9.7 | -- | -- | -- | -- | 0 | 0.0 | 1 | 0.3 | 9 | 2.7 | 20 | 6.2 | 20 | 15.4 |
| Education |  |  |  |  |  |  |  |  |  |  |  |  |  |  |  |  |  |  |  |  |
| 8th grade or less | 4 | 0.5 | 7 | 0.9 | 4 | 0.6 | -- | -- | 3 | 0.8 | 2 | 0.6 | 4 | 0.9 | 3 | 0.7 | 2 | 0.5 | 1 | 0.6 |
| Some high school | 137 | 18.7 | 40 | 5.3 | 29 | 4.3 | -- | -- | 111 | 28.8 | 23 | 6.7 | 25 | 5.3 | 25 | 6.2 | 15 | 3.7 | 7 | 4.5 |
| High school graduate | 184 | 25.2 | 123 | 16.4 | 105 | 15.4 | -- | -- | 115 | 29.8 | 71 | 20.7 | 76 | 16.2 | 65 | 16.2 | 67 | 16.5 | 18 | 11.5 |
| GED | 31 | 4.2 | 47 | 6.3 | 43 | 6.3 | -- | -- | 14 | 3.6 | 21 | 6.1 | 32 | 6.8 | 26 | 6.5 | 23 | 5.7 | 5 | 3.2 |
| Some vocational/ technical school | 18 | 2.5 | 8 | 1.1 | 11 | 1.6 | -- | -- | 8 | 2.1 | 7 | 2.0 | 10 | 2.1 | 3 | 0.7 | 6 | 1.5 | 3 | 1.9 |
| Completed vocational /technical school | 16 | 2.2 | 26 | 3.5 | 41 | 6.0 | -- | -- | 5 | 1.3 | 9 | 2.6 | 14 | 3.0 | 25 | 6.2 | 19 | 4.7 | 11 | 7.1 |
| Some college | 280 | 38.3 | 251 | 33.5 | 192 | 28.2 | -- | -- | 128 | 33.2 | 151 | 44.0 | 158 | 33.6 | 121 | 30.2 | 119 | 29.4 | 46 | 29.5 |
| AA degree (2-year college degree) | 31 | 4.2 | 70 | 9.3 | 62 | 9.1 | -- | -- | 2 | 0.5 | 32 | 9.3 | 47 | 10.0 | 28 | 7.0 | 36 | 8.9 | 18 | 11.5 |
| BA or BS (4-year college degree) | 19 | 2.6 | 123 | 16.4 | 121 | 17.8 | -- | -- | 0 | 0 | 22 | 6.4 | 74 | 15.7 | 69 | 17.2 | 73 | 18.0 | 25 | 16.0 |
| Some graduate or professional school | 7 | 1.0 | 28 | 3.7 | 22 | 3.2 | -- | -- | 0 | 0 | 5 | 1.5 | 19 | 4.0 | 20 | 5.0 | 9 | 2.2 | 4 | 2.6 |
| Completed graduate/professional school | 4 | 0.5 | 26 | 3.5 | 51 | 7.5 | -- | -- | 0 | 0 | 0 | 0 | 11 | 2.3 | 16 | 4.0 | 36 | 8.9 | 18 | 11.5 |
| Employment |  |  |  |  |  |  |  |  |  |  |  |  |  |  |  |  |  |  |  |  |
| No current paid employment | 147 | 9.7 |  | 20.1 | 112 | 15.0 | -- | -- | 97 | 14.2 | 94 | 24.3 | 51 | 14.8 | 66 | 14.0 | 67 | 16.7 | 62 | 15.3 |
| Yes, part time | 209 | 13.8 |  | 28.5 | 76 | 10.1 | -- | -- | 78 | 11.5 | 144 | 37.2 | 67 | 19.5 | 53 | 11.3 | 35 | 8.7 | 40 | 9.9 |
| Yes, full time | 373 | 24.6 |  | 50.9 | 555 | 74.1 | -- | -- | 501 | 73.6 | 148 | 38.2 | 224 | 65.1 | 346 | 73.6 | 297 | 74.1 | 300 | 74.1 |
| Temporarily laid off | 3 | 0.2 |  | 0.4 | 2 | 0.3 | -- | -- | 5 | 0.7 | 0 | 0 | 2 | 0.6 | 2 | 0.4 | 1 | 0.2 | 3 | 0.7 |
| Assistantship or fellowship | 1 | 0.1 |  | 0.1 | 4 | 0.5 | -- | -- | 0 | 0 | 1 | 0.3 | 0 | 0 | 3 | 0.6 | 0 | 0 | 0 | 0 |

Table S6.2

*Prevalences of other lifecourse events in AHB across waves and age bins*

|  | Wave 4 | | Wave 5 | | Wave 6 | | Wave 7 | | -- | | Age bin 21-23 | | Age bin 24-26 | | Age bin 27-30 | | Age bin 31-34 | | Age bin 35-39 | |
| --- | --- | --- | --- | --- | --- | --- | --- | --- | --- | --- | --- | --- | --- | --- | --- | --- | --- | --- | --- | --- |
|  | N | % | n | % | n | % | n | % | -- | -- | n | % | N | % | n | % | n | % | n | % |
| Marital status |  |  |  |  |  |  |  |  |  |  |  |  |  |  |  |  |  |  |  |  |
| Never married or divorced | -- | -- | -- | -- | -- | -- | -- | -- | -- | -- | -- | -- | -- | -- | -- | -- | -- | -- |  |  |
| never married | 446 | 94.7 | 297 | 65.1 | 117 | 28.7 | 72 | 18.8 | -- | -- | 431 | 94.3 | 293 | 65.0 | 112 | 27.7 | 51 | 19.4 | 7 | 5.3 |
| Divorced | 2 | 0.4 | 3 | 0.7 | 16 | 3.9 | 17 | 4.5 | -- | -- | 2 | 0.4 | 3 | 0.7 | 16 | 4.0 | 11 | 4.2 | 6 | 4.5 |
| Separated | 0 | 0 | 3 | 0.7 | 3 | 0.7 | 4 | 1 | -- | -- | 0 | 0 | 3 | 0.7 | 3 | 0.7 | 4 | 1.5 | 0 | 0.0 |
| Engaged | 0 | 0 | 0 | 0 | 20 | 4.9 | 6 | 1.6 | -- | -- | -- | -- | 0 | 0.0 | 26 | 6.4 | 3 | 1.1 | 26 | 19.7 |
| Married | 23 | 4.9 | 153 | 33.6 | 252 | 61.8 | 280 | 73.3 | -- | -- | 24 | 5.3 | 152 | 33.7 | 248 | 61.2 | 192 | 73.0 | 92 | 69.7 |
| Number of children |  |  |  |  |  |  |  |  |  |  |  |  |  |  |  |  |  |  |  |  |
| 0 | 454 | 96.4 | 405 | 88.8 | 263 | 64.5 | 140 | 36.6 | -- | -- | 441 | 96.5 | 402 | 89.1 | 263 | 64.9 | 100 | 38.0 | 47 | 35.6 |
| 1 | 15 | 3.2 | 41 | 9.0 | 82 | 20.1 | 68 | 17.8 | -- | -- | 14 | 3.1 | 39 | 8.6 | 80 | 19.8 | 48 | 18.3 | 25 | 18.9 |
| 2 | 2 | 0.4 | 10 | 2.2 | 56 | 13.7 | 127 | 33.2 | -- | -- | 2 | 0.4 | 10 | 2.2 | 55 | 13.6 | 84 | 31.9 | 44 | 33.3 |
| 3 | 0 | 0 | 0 | 0 | 6 | 1.5 | 36 | 9.4 | -- | -- | 0 | 0 | 0 | 0 | 6 | 1.5 | 23 | 8.7 | 13 | 9.8 |
| 4 | 0 | 0 | 0 | 0 | 1 | 0.2 | 9 | 2.4 | -- | -- | 0 | 0 | 0 | 0 | 1 | 0.2 | 6 | 2.3 | 3 | 2.3 |
| 5 | 0 | 0 | 0 | 0 | 0 | 0 | 2 | 0.5 | -- | -- | 0 | 0 | 0 | 0 | 0 | 0 | 2 | 0.8 | 0 | 0 |
| Education |  |  |  |  |  |  |  |  |  |  |  |  |  |  |  |  |  |  |  |  |
| GED | -- | -- | -- | -- | 0 | 0 | 0 | 0 | -- | -- | -- | -- | -- | -- | 0 | 0 | 0 | 0 | 0 | 0 |
| High School diploma | -- | -- | -- | -- | 65 | 15.9 | 46 | 12.0 | -- | -- | -- | -- | -- | -- | 60 | 15.1 | 33 | 12.6 | 18 | 13.7 |
| Vocational/technical diploma | -- | -- | -- | -- | 1 | 0.2 | -- | -- | -- | -- | -- | -- | -- | -- | 1 | 0.3 |  |  |  |  |
| Associate degree | -- | -- | -- | -- | 14 | 3.4 | 13 | 3.4 | -- | -- | -- | -- | -- | -- | 13 | 3.3 | 12 | 4.6 | 2 | 1.5 |
| R.N. diploma | -- | -- | -- | -- | 1 | 0.2 | 2 | 0.5 | -- | -- | -- | -- | -- | -- | 1 | 0.3 |  |  | 2 | 1.5 |
| Bachelor degree | -- | -- | -- | -- | 234 | 57.4 | 200 | 52.4 | -- | -- | -- | -- | -- | -- | 230 | 57.8 | 145 | 55.6 | 59 | 45.0 |
| Master degree | -- | -- | -- | -- | 65 | 15.9 | 90 | 23.6 | -- | -- | -- | -- | -- | -- | 65 | 16.3 | 56 | 21.5 | 34 | 26.0 |
| Doctorate (M.D., Ph.D., J.D., etc.) | -- | -- | -- | -- | 28 | 6.9 | 31 | 8.1 | -- | -- | -- | -- | -- | -- | 28 | 7.0 | 15 | 5.7 | 16 | 12.2 |
| Full-time employment |  |  |  |  |  |  |  |  |  |  |  |  |  |  |  |  |  |  |  |  |
| No | -- | -- | -- | -- | 106 | 26.0 | 107 | 28.0 | -- | -- | -- | -- | -- | -- | 105 | 26.0 | 79 | 30.0 | 29 | 22.0 |
| Yes | -- | -- | -- | -- | 301 | 74.0 | 275 | 72.0 | -- | -- | -- | -- | -- | -- | 292 | 73.6 | 182 | 69.7 | 102 | 77.9 |

**Online Supplement S7:**

**Supplemental AFDP models ruling out confounding effects of parenthood and marital engagement as potential explanations for findings**

To address parenthood as a potential confounder, we classified participants on the basis of parenthood transitions between time points (for descriptives, see Table S7.1below). We then re-estimated our primary model, again testing our key contrast of interest between those who became married versus remained unmarried between time points; except in this variation, those who had become parents at a given time point (i.e., transition from non-parent at the preceding time point to parent at the current time point) were excluded from the contrast of those who became married versus remained unmarried at that time point. As reported in Table S7.2 below, this adjustment for parenthood yielded slightly more robust evidence for the current study’s primary conclusion of age-related declines in the magnitude of marriage effects on problem-drinking reductions.

To address engagement as a potential confounder, we classified participants on the basis of transitions in and out of engagement between time points (for descriptives, see Table S7.1 below), and then we re-estimated our model with those who had transitioned from engaged to married or single to engaged at a given time point excluded from key the contrast of those who became married versus remained unmarried at that time point. As reported in Table S7.3 below, this adjustment for engagement yielded results that were highly similar to those of our primary analyses.

Table S7.1

Descriptive information regarding other lifecourse transitions between time points in the AFDP sample

|  | Age bin  17-20 | Age bin  21-23 | Age bin  24-26 | Age bin  27-30 | Age bin  31-34 | Age bin  35-39 |
| --- | --- | --- | --- | --- | --- | --- |
| *N* | 324 | 250 | 330 | 300 | 281 | 65 |
| Became Married | 0 (0%) | 16 (2.8%) | 84 (14.6%) | 104 (18.0%) | 89 (15.4%) | 13 (2.3%) |
| Post-Marriage | 0 (0%) | 0 (0%) | 0 (0%) | 38 (6.6%) | 82 (14.2%) | 28 (4.9%) |
| Became Married excluding those who became parents | 0 (0%) | 8 (1.4%) | 56 (9.7%) | 57 (9.9%) | 47 (8.1%) | 9 (1.6%) |
| Post-Marriage excluding those who became parents | 0 (0%) | 0 (0%) | 0 (0%) | 27 (4.7%) | 50 (8.7%) | 26 (4.5%) |
| Became Married excluding those engaged at preceding time point | 0 (0%) | 16 (2.8%) | 84 (14.6%) | 99 (17.2%) | 79 (13.7%) | 11 (1.9%) |
| Post-Marriage excluding those engaged at preceding time point | 0 (0%) | 0 (0%) | 0 (0%) | 37 (6.4%) | 81 (14.0%) | 28 (4.9%) |
| Became Parent | 0 (0%) | 10 (1.7%) | 43 (7.5%) | 78 (13.5%) | 85 (14.7%) | 6 (1.0%) |
| Post-Parenthood | 0 (0%) | 4 (.7%) | 29 (5.0%) | 53 (9.2%) | 78 (13.5%) | 32 (5.5%) |
| Became Engaged | 0 (0%) | 6 (1.0%) | 15 (2.6%) | 18 (3.1%) | 6 (1.0%) | 2 (.3%) |
| Remained Married | 0 (0%) | 0 (0%) | 0 (0%) | 22 (3.8%) | 52 (9.0%) | 21 (3.6%) |
| Became Divorced | 0 (0%) | 0 (0%) | 0 (0%) | 5 (.9%) | 8 (1.4%) | 6 (1.0%) |
| Engaged to Married | 0 (0%) | 0 (0%) | 0 (0%) | 6 (1.0%) | 9 (1.6%) | 2 (.3%) |
| Remained not full-time employed | 0 (0%) | 16 (2.8%) | 43 (7.5%) | 23 (4.0%) | 26 (4.5%) | 4 (.7%) |
| Became Full-time employed | 0 (0%) | 50 (8.7%) | 105 (18.2%) | 40 (6.9%) | 32 (5.5%) | 5 (.9%) |
| Remained Full-time employed | 0 (0%) | 17 (2.9%) | 97 (16.8%) | 151 (26.2%) | 149 (25.8%) | 43 (7.5%) |
| Became not full-time employed | 0 (0%) | 0 (0%) | 0 (0%) | 3 (.5%) | 18 (3.1%) | 33 (5.7%) |

Table S7.2

Supplemental AFDP model ruling out confounding effects of parenthood as potential explanations for findings

|  | Estimates | p-value |
| --- | --- | --- |
| Effects on age 17-20 problem drinking |  |  |
| Sex (0=male; 1=female) | -0.862 | 0.000 |
| Familial AUD (0=negative; 1=positive) | 0.451 | 0.022 |
| Effects on age 21-23 problem drinking |  |  |
| **Became married by age 21-23** | **-1.316** | **0.136** |
| Sex (0=male; 1=female) | -0.822 | 0.000 |
| Familial AUD (0=negative; 1=positive) | 0.597 | 0.000 |
| Effects on age 24-26 problem drinking |  |  |
| **Became married by age 24-26** | **-0.973** | **0.001** |
| Sex (0=male; 1=female) | -0.277 | 0.284 |
| Familial AUD (0=negative; 1=positive) | 0.221 | 0.306 |
| Effects on age 27-30 problem drinking |  |  |
| **Became married by age 27-30** | **0.163** | **0.599** |
| Post-marriage by age 27-30 | 0.548 | 0.185 |
| Sex (0=male; 1=female) | -0.933 | 0.000 |
| Familial AUD (0=negative; 1=positive) | 0.566 | 0.012 |
| Effects on age 31-34 problem drinking |  |  |
| **Became married by age 31-34** | **-0.249** | **0.394** |
| Post-marriage by age 31-34 | 0.074 | 0.839 |
| Sex (0=male; 1=female) | -0.732 | 0.009 |
| Familial AUD (0=negative; 1=positive) | 0.501 | 0.059 |
| Effects on age 35-39 problem drinking |  |  |
| **Became married by age 35-39** | **0.818** | **0.286** |
| Post-marriage by age 35-39 | -0.394 | 0.582 |
| Sex (0=male; 1=female) | -0.090 | 0.909 |
| Familial AUD (0=negative; 1=positive) | 0.111 | 0.883 |
| **Wald χ^2^ *omnibus* test of age moderation of the “became married” effect** | **χ^2^(4)=11.86 (p=0.019)** | |
| **Wald χ^2^ *linear* test of age moderation of the “became married” effect** | **χ^2^(1)=6.388 (p=0.012)** | |
| Growth factor means |  |  |
| Intercept factor | 0.538 | 0.052 |
| Slope factor | -1.802 | 0.126 |
| Growth factor covariance |  |  |
| Intercept factor with slope factor | 0.883 | 0.413 |
| Intercept factor loadings |  |  |
| Age 17-20 problem drinking | @1 | -- |
| Age 21-23 problem drinking | @1 | -- |
| Age 24-26 problem drinking | @1 | -- |
| Age 27-30 problem drinking | @1 | -- |
| Age 31-34 problem drinking | @1 | -- |
| Age 35-39 problem drinking | @1 | -- |
| Slope factor loadings |  |  |
| Age 17-20 problem drinking | @0 | -- |
| Age 21-23 problem drinking | @0.17 | -- |
| Age 24-26 problem drinking | @0.33 | -- |
| Age 27-30 problem drinking | @0.50 | -- |
| Age 31-34 problem drinking | @0.69 | -- |
| Age 35-39 problem drinking | @1 | -- |

Table S7.3

Supplemental AFDP model ruling out confounding effects of marital engagement as potential explanations for findings

|  | Estimates | p-value |
| --- | --- | --- |
| Effects on age 17-20 problem drinking |  |  |
| Sex (0=male; 1=female) | -0.882 | 0.000 |
| Familial AUD (0=negative; 1=positive) | 0.448 | 0.024 |
| Effects on age 21-23 problem drinking |  |  |
| **Became married by age 21-23** | **-1.700** | **0.004** |
| Sex (0=male; 1=female) | -0.836 | 0.000 |
| Familial AUD (0=negative; 1=positive) | 0.569 | 0.001 |
| Effects on age 24-26 problem drinking |  |  |
| **Became married by age 24-26** | **-1.188** | **0.000** |
| Sex (0=male; 1=female) | -0.301 | 0.238 |
| Familial AUD (0=negative; 1=positive) | 0.240 | 0.279 |
| Effects on age 27-30 problem drinking |  |  |
| **Became married by age 27-30** | **-0.534** | **0.048** |
| Post-marriage by age 27-30 | -0.261 | 0.544 |
| Sex (0=male; 1=female) | -0.809 | 0.002 |
| Familial AUD (0=negative; 1=positive) | 0.570 | 0.011 |
| Effects on age 31-34 problem drinking |  |  |
| **Became married by age 31-34** | **-0.629** | **0.031** |
| Post-marriage by age 31-34 | -0.336 | 0.239 |
| Sex (0=male; 1=female) | -0.798 | 0.004 |
| Familial AUD (0=negative; 1=positive) | 0.457 | 0.095 |
| Effects on age 35-39 problem drinking |  |  |
| **Became married by age 35-39** | **0.004** | **0.997** |
| Post-marriage by age 35-39 | -0.884 | 0.222 |
| Sex (0=male; 1=female) | -0.138 | 0.859 |
| Familial AUD (0=negative; 1=positive) | -0.065 | 0.937 |
| **Wald χ^2^ *omnibus* test of age moderation of the “became married” effect** | **χ^2^(4)=6.242 (p=0.182)** | |
| **Wald χ^2^ *linear* test of age moderation of the “became married” effect** | **χ^2^(1)=5.375 (p=0.020)** | |
| Growth factor means |  |  |
| Intercept factor |  |  |
| Slope factor |  |  |
| Growth factor covariance |  |  |
| Intercept factor with slope factor |  |  |
| Intercept factor loadings |  |  |
| Age 17-20 problem drinking | @1 | -- |
| Age 21-23 problem drinking | @1 | -- |
| Age 24-26 problem drinking | @1 | -- |
| Age 27-30 problem drinking | @1 | -- |
| Age 31-34 problem drinking | @1 | -- |
| Age 35-39 problem drinking | @1 | -- |
| Slope factor loadings |  |  |
| Age 17-20 problem drinking | @0 | -- |
| Age 21-23 problem drinking | @0.17 | -- |
| Age 24-26 problem drinking | @0.33 | -- |
| Age 27-30 problem drinking | @0.50 | -- |
| Age 31-34 problem drinking | @0.69 | -- |
| Age 35-39 problem drinking | @1 | -- |
